# Breaking the 1250 nm Barrier: A Computational Approach to Light Upconversion via Triplet–Triplet Annihilation in the Silica Telecom Band

**DOI:** 10.1021/acsaem.5c04014

**Published:** 2026-02-12

**Authors:** Jenny G. Vitillo

**Affiliations:** Department of Science and High Technology and INSTM, Università degli Studi dell’Insubria, Via Valleggio 9, 22100 Como, Italy

**Keywords:** triplet−triplet annihilation, light upconversion, TTA-UC, telecom NIR, silica nanofibers, density functional theory

## Abstract

The 1250–1675 nm region is the spectral range exploited in modern telecommunications and quantum networks because of the high transparency of silica-based nanofibers. Nevertheless, the low efficiency of detectors and sensors in that region requires light upconversion (UC). This is achieved with inorganic dopants, often based on rare-earth elements or metal nanoparticles that are characterized by a low chemical flexibility besides sustainability issues. Triplet–triplet annihilation upconversion (TTA-UC) is a process that exploits as emitters organic molecules that would remove or mitigate these limitations. Nevertheless, no TTA-UC emitters have been reported to be able to absorb in the telecom region and very few in the region at wavelengths longer than 1000 nm. Here, we used Kohn–Sham density functional calculations to tune the triplet energy of tetracene, the parent molecule of a class of emitters for infrared light UC. We highlight three organic molecules, each with an existing synthetic procedure, as promising near-infrared (NIR) TTA-UC annihilators. Additionally, we computationally predict 5,12-bis­(*N*,*N*-diaminobenzene)­tetracene as an emitter for TTA-UC of light beyond 1250 nm. These findings pave the way for the design and development of organic molecules for upconverting the telecom-band NIR light.

## Graphical abstract

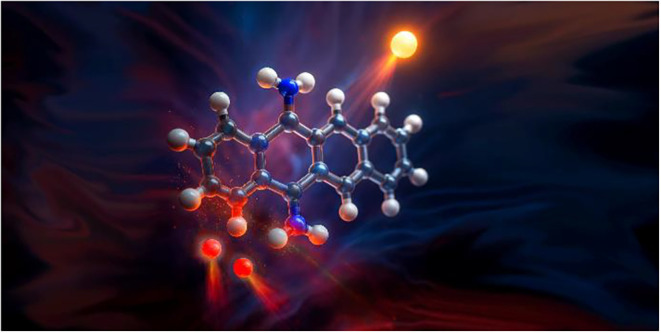

## 1 Introduction

The conversion of low-energy photons to higher-energy photons, known as photon upconversion, has emerged as a critical tool in photonics and quantum technologies.^1^ Among the various wavelengths, the region between 1250 and 1675 nm holds particular significance due to its compatibility with the existing optical fiber infrastructure, eye-safe properties, and utility in long-distance communications and quantum networks.^2–4^ However, many high-performance photodetectors and quantum devices exhibit limited sensitivity in the short-wave infrared (SWIR or NIR-II, 1000–2500 nm) region, prompting the use of upconversion strategies to convert these longer wavelengths into the visible or NIR-I ranges where detection is more efficient.^5^

Silica-based optical fibers exhibit minimal transmission loss and low chromatic dispersion in three key spectral windows: 800–900 nm, 1250–1350 nm, and 1530–1675 nm,^2–4,6^ with the second and third regions that can be joined using fibers with low or negligible hydroxyl content.^4^ For their transparency, these regions have been widely exploited in telecommunications and quantum networks.^4^ The two lowest regions are characterized by the lowest transmission losses, making them the most efficient ranges for modern optical communication. This property enables low-attenuation signal transmission over long distances, which is essential for long-haul data links. Despite this advantage, direct detection of NIR-II photons remains challenging due to the limited efficiency of conventional infrared detectors compared to their visible-light counterparts,^5^ that necessitate the use of fiber amplifiers based on rare-earth metals, such as erbium, praseodymium, or thulium.^3,4^ Upconversion could address this limitation by shifting photons to shorter wavelengths (e.g., 700–1000 nm), where silicon-based detectors achieve superior sensitivity and lower noise.

The 1250–1675 nm region not only underpins modern fiber-optic telecommunications but also lies within a broad atmospheric-transparency window.^7^ Operating Laser Imaging Detection and Ranging (LiDAR) systems in this NIR-II range can therefore reduce scattering and atmospheric losses compared with visible and NIR-I wavelengths, improving long-range performance. However, most imaging and detection hardware still relies on visible or NIR-I sensing, making spectral upconversion essential to exploit fully NIR-II excitation while retaining compatibility with existing detector technologies.^8,9^ As for energy harvesting, upconversion of sub-bandgap photons represents a promising route to improve photovoltaic efficiency by converting otherwise unutilized low-energy photons into wavelengths that can be absorbed by the solar cell.^10^ Photocatalysis and photoredox catalysis would also significantly benefits from the exploitation of NIR-II photons through upconversion.^5^

Photon upconversion in the NIR-II has been investigated through various approaches, mainly based on rare-earth metals.^1^ Upconversion has been observed in LiYF_4_ nanocrystals doped with Er^3+^ under 1490 nm excitation.^1^ Moreover, the incorporation of gold plasmonic nanostructures has been shown to enhance the upconversion efficiency in TiO_2_ matrices doped with Er^3+^.^1^ Despite their intrinsic robustness, such systems allow only restricted manipulation of excited-state energetics and depend on elements with a non-negligible environmental impact and constrained global availability. Organic upconverting architectures provide a viable route to overcome these limitations, enabling fine molecular tailoring together with more sustainable and economical manufacturing. Triplet–triplet annihilation upconversion (TTA-UC)^11–16^ is a photophysical process in which two low-energy photons are converted into one higher-energy photon through a mechanism that typically involves a sensitizer that absorbs visible or NIR-I light and transfers its energy to an organic molecule, the annihilator, which subsequently undergoes TTA to emit a photon of higher energy. TTA-UC systems that can operate in the near-infrared (NIR) region are underexplored.^5,12^ To date, no specific reports are available on organic molecules capable of achieving TTA-UC upon excitation beyond 1148 nm,^12,13,17–20^ with the largest wavelength (1148 nm) absorbed by 5,11-bis­(triethylsilylethynyl)­anthradithiophene (TES-ADT, see Scheme 1).^21,22^ The lack of annihilators suitable for TTA-UC in the telecom band represents a critical limitation.

**Scheme 1:**
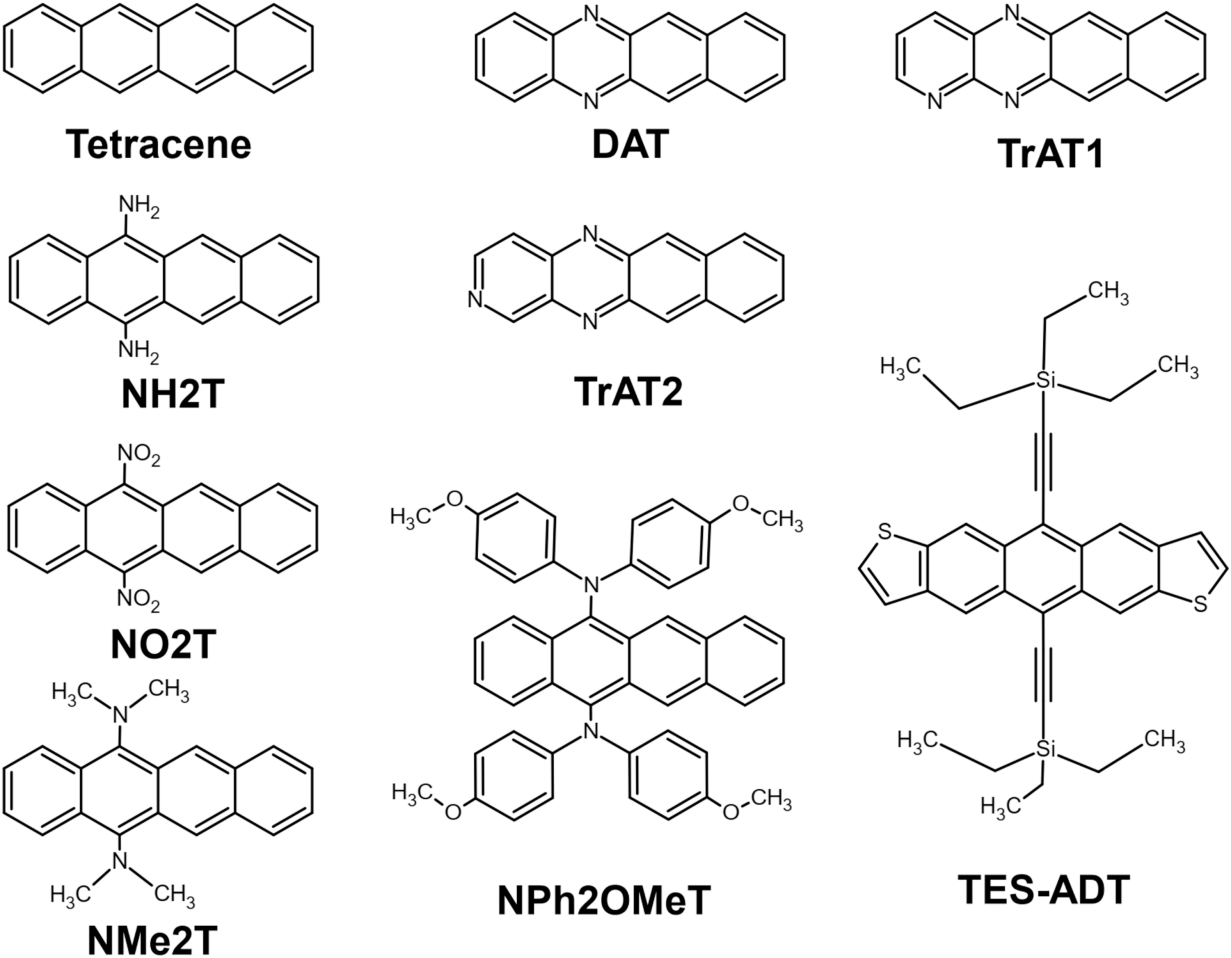
Tetracene Derivatives Considered in This Study and the State-of-the-Art TES-ADT

To address this, here a new molecule is computationally designed and tailored to operate in the transparency window of silica optical fibers below 1250 nm, featuring a triplet energy lower than previously reported annihilators. Kohn–Sham Density Functional methods (KS-DFT) are used here to tune the triplet energy of tetracene derivatives through targeted functionalization and heteroatom substitution, exploiting the known impact of nitrogen-containing groups on the electronic gaps of organic molecules.^23,24^ We then compared the electronic properties of the parent molecule tetracene and of TES-ADT with three azatetracenes—5,12-diazatetracene (DAT),^25^ 1,5,12-triazatetracene (TrAT1),^25^ and 2,5,12-triazatetracene (TrAT2)^25^; three 5,12-diamino-tetracenes—5,12-bis­(*N*,*N*-diamino)­tetracene (NH2T), 5,12-bis­(*N*,*N*-dimethylamino)­tetracene (NMe2T),^26^ and 5,12-bis­(*N*,*N*-dianisylamino)­tetracene (NPh2OMeT);^26^ and 5,12-bis­(N,N-dinitro)­tetracene (NO2T, see Scheme 1). All molecules in this set have been previously reported experimentally, except for compounds NH2T and NO2T. Nevertheless, their structural simplicity and analogy to previously reported tetracene derivatives suggest that plausible synthetic routes could be envisaged.

In the last part of the study, being the TTA-UC process based on the collision of two annihilators, we have investigated the dimers of tetracene and of the molecule showing the smallest triplet energy, NH2T, with the aim to obtain their dimer formation energies and verify how the geometrical and electronic properties change upon dimerization and functionalization.

This study enlarges the set of emitters for NIR TTA-UC and lays the foundations for the use of TTA for the upconversion of photons having energy in the silica telecom band. In fact, while the efficiency of a complete TTA-UC system is ultimately dictated by a complex interplay of factors (including aggregation, excitonic coupling, nonradiative decay channels, and sensitizer-annihilator interactions), the present study specifically targets the identification of annihilators capable of accessing the telecom-relevant triplet energy regime, which was unexplored even at the molecular design level.

## 2 Computational Methods

All calculations were performed using the B3LYP^27,28^ density functional including the D3 version of Grimme’s dispersion with the Becke-Johnson damping (BJ)^29^ as implemented in *Gaussian 16* program.^30^ It was used in combination with the triple-ζ basis sets def2-TZVP.^31,32^ A (99,590) pruned grid was used (i.e., 99 radial points and 590 angular points per radial point), corresponding to the grid = ultrafine option.

Geometry optimization was carried out by means of the Berny optimization algorithm with an analytical gradient and default convergence thresholds. Unscaled, harmonic vibrational frequencies were computed analytically to confirm that all reported structures corresponded to minima. Charge and spin densities were obtained using Charge Model 5 (CM5)^33^ and Hirshfeld population analysis,^34^ respectively.

Time-dependent (TD) density functional theory in the frame of the linear response theory^35^ is the most common approach for the study of molecular electronic excited states.^36,37^ TD-DFT calculations were performed using the B3LYP functional, which has been shown to provide a reliable and balanced description of excited-state energetics in conjugated organic chromophores (see also refs 38,39 ). The electronic absorption (emission) spectra were computed by single-point calculations on the ground state (first excited singlet state) optimized structures by considering the first 100 excitations. All the calculations have been performed in the gas phase, besides some selected calculations where a polarizable continuum dichloromethane model was also adopted (ε = 8.93)^40^ to allow the comparison with the experiments. A Gaussian broadening was applied to the TD-DFT excitations using a standard deviation of 0.3 eV for the plotting of the UV spectra. No scaling factors have been adopted. Visualization of the computed spectra and of the total density maps was performed using GaussView 6.0.

The vertical excitation energies (Δ*E*_vert_) for S_0_ → S_1_, S_0_ → T_1_, S_0_ → T_2_, and S_0_ → Q_1_, as well as the electronic adiabatic excitation energies (Δ*E*_adia_) for S_0_ → S_1_, S_0_ → T_1_, and S_0_ → Q_1_, were obtained as the energy difference between the ground-state energy and the energy of the excited state computed through single points and geometry optimizations, respectively. No difference was observed among geometry optimizations for S_1_ including the first 5, 7, and 100 excitations in an initial assessment on tetracene. For this reason, only the first ten excitations were considered in the geometry optimization. ZPVE-corrected adiabatic excitation energies were also obtained (Δ*E*_adiaZPVE_). For T_1_ and Q_1_, only the geometries optimized at the unrestricted DFT level (without TD) are reported in the following. During TD-DFT geometry optimizations of the T_1_ state, the lowest triplet excitation energy (root 1) was occasionally found to converge to negative values. Such behavior is a recognized signature of triplet instabilities in linear response TD-DFT.^41,42^ To ensure reliable geometries, we therefore optimized T_1_ at the unrestricted DFT level, which provided well-behaved stationary points. For the monomers, S_1_, including both singlet and triplet states, yielded the same results as singlet-only optimizations. In contrast, triplet instabilities were observed for the dimer, so the TD optimization was carried out excluding triplet states.

All energetic data for the dimers have been corrected for the basis set superposition error (BSSE) following the a posteriori method proposed by Boys and Bernardi,^43^ as implemented in *Gaussian 16*. The BSSE-corrected values are indicated by the superscript c and were obtained from the computed *Y* values as *Y* ^c^ = *Y* + BSSE.

The radiative decay rate *k*_rad_ of the first excited singlet state was estimated using the Einstein coefficient for spontaneous emission. Following the definition commonly adopted in refs 44,45 , *k*_rad_ was first evaluated using the vertical excitation energy and transition dipole moment associated with the S_0_ → S_1_ transition, i.e., at the ground-state optimized geometry (*k*_rad_^S_0_→S_1_^). In addition, the radiative decay rate corresponding to the physically rigorous emission process, *k*_rad_^S_1_→S_0_^, was explicitly computed using the vertical emission energy and transition dipole moment evaluated at the optimized S_1_ geometry

1 $$A_{S1\to S0}=k_{\mathrm{rad}}^{S1\to S0}=\frac{{\left(\Delta E_{\mathrm{vert}}^{\prime }\right)}^{3}\cdot {\left|\mu_{S1\to S0}\right|}^{2}}{3\cdot \pi \cdot \varepsilon_{0}\cdot \hbar^{4}\cdot c^{3}}$$

where ε_0_, *c*, and ℏ represent the vacuum permittivity, the speed of light, and the reduced Planck constant, respectively, while Δ*E*_vert_^′^ is the vertical energy corresponding to the S_1_ → S_0_ transition computed on the S_1_ optimized structure and μ_S1→S0_ is the corresponding transition electric dipole moment. Comparison between the two definitions allows quantification of the deviation introduced by neglecting excited-state relaxation effects, which are ignored in the standard S_0_ → S_1_-based approximation but may become relevant for quantitative predictions.

The planarity of the π-conjugated core can be affected by functionalization. The planarity was then quantified through an automated geometric workflow implemented in Python 3.10. Using a custom script,^46^ we first selected all heavy atoms (C, N, S) comprising the central aromatic system. The geometric centroid of these atoms served as the reference point for subsequent plane-fitting analysis. The mean absolute orthogonal displacement of core atoms from this plane provided the planarity metric (*d*_avg_), calculated as

$$d_{\mathrm{avg}}=\frac{1}{N}\sum_{l=1}^{N}\left|\left(r_{i}-r_{0}\right)\cdot \mathrm{n̂}\right|$$

where *r*_*i*_ represents atomic positions, *r*_0_ the centroid, and *n̂* the unit normal vector.

To evaluate how the functional group affects molecular size and thus the potential packing and TTA efficiency, using the same script, we characterized the three-dimensional molecular envelope by projecting all atomic coordinates onto the normal vector axis. The maximum (*R*_above_) and minimum (*R*_below_) projection values relative to the centroid defined the vertical dimensions and were obtained using the formulas

$$R_{\mathrm{above}}=\max\{\left(r_{i}-r_{0}\right)\cdot \mathrm{n̂}\}$$

$$R_{\mathrm{below}}=\left|\min\left\{\left(r_{i}-r_{0}\right)\cdot \mathrm{n̂}\right\}\right|$$

The aromatic character of the tetracene derivatives was evaluated by using nucleus-independent chemical shift (NICS) calculations. NICS probes were placed at the ring center (NICS(0)) and 1 Å above the ring center (NICS(1)) for each aromatic ring, and the magnetic shielding tensors were evaluated at the optimized S_0_ geometries. As the primary aromaticity descriptor, NICS(1)_*zz* values were employed, defined as the *zz* component of the magnetic shielding tensor calculated at a ghost atom (Bq) positioned 1 Å above the ring center with the *z* axis oriented perpendicular to the molecular plane. This component selectively probes π-electron ring currents and minimizes σ-electron contributions and is therefore widely regarded as the most reliable NICS-based measure of aromaticity in planar conjugated systems.^47,48^

Natural transition orbitals (NTOs) were computed for selected singlet and triplet excitations using ground-state optimized geometries. The NTOs were obtained from the one-particle transition density matrix corresponding to each electronic transition and represent the most compact hole–particle description of the excitation. For each transition, only the leading NTO pairs with the largest weights (>0.1) were analyzed and visualized, as they capture the dominant electronic character of the excitation.

## 3 Results and Discussion

### 3.1 Monomers

The geometrical properties of the monomers, optimized in the ground (S_0_) and excited (S_1_, T_1_), states, are summarized in Table S1. These data indicate that the different substituents do not significantly perturb the planarity of the tetracene core, with deviations below 0.025 Å in S_0_ and below 0.11 Å in the optimized excited states. The vertical (geometrical) dimensions of the molecules, as given by *R*_above_ and *R*_below_, indicate that only NMe2T, NPh2OMeT, and NO2T increase the molecular dimensions compared to the parent compound (see Table S1). These size differences may influence the intermolecular packing in dimers and, consequently, the efficiency of the TTA.

We also evaluated whether the functional groups induce a significant redistribution of the charge density on the tetracene scaffold. All tetracenes showed an increase (a decrease in modulus) in the charge on the tetracene core with respect to the parent compound, independently of the population analysis employed (see Table S1). The largest increase is observed for NO2T and NPh2OMeT (*q*_CM5_ = −0.62 and −0.77 au to be compared with −1.16 au obtained for tetracene). These values remain essentially unchanged upon electronic excitation.

The effect of functionalization on the electronic transitions is markedly more pronounced, in terms of both orbital contributions and excitation energies (see Tables 1 and 2). The main orbitals involved are reported in Table 1 and Section S3.1 of the Supporting Information. For the S_0_ → T_1_ and S_0_ → S_1_ transitions, the dominant contribution arises from HOMO (H) → LUMO (L) excitation. This is illustrated by the difference-density maps shown in Figures S2 and S3 that are highly similar for both families of transitions: the regions of decreased electron density (orange) correspond predominantly to the HOMO, while the regions of increased density after excitation (light blue) reflect the LUMO distribution. For the S_0_ → T_2_ transition, however, the orbital character strongly depends on the nature of the substituent (see Figure S4), although the H–1 → L contribution remains predominant in most cases. The only significant exception is observed for NPh2OMeT, which exhibits additional contributions from H-2 → L in the S_0_ → T_1_ transition and shows a distinctly different orbital nature in S_0_ → T_2_.

**Table 1:** Major Contributions (Coefficients >0.20) to the Vertical Excitations for Tetracene Derivatives (TD-B3LYP/def2-TZVP)^a^

	tetracene	NH2T	NMe2T	NPh2OMeT	NO2T	DAT	TrAT1	TrAT2	TES-ADT
S_0_ → T_1_	H → L	H → L	H → L	H → L (0.60)	H → L	H → L	H → L	H → L	H → L
				H–2 → L (0.38)					
S_0_ → S_1_	H → L	H → L	H → L	H → L	H → L	H → L	H → L	H → L	H → L
S_0_ → T_2_	H–1 → L (0.53)	H–1 → L (0.49)	H–3 → L (0.54)	H–2 → L (0.58)	H–1 → L (0.42)	H–1 → L	H–1 → L	H–1 → L	H–1 → L (0.59)
	H → L + 1 (−0.45)	H → L + 1 (−0.48)	H → L+1 (0.38)	H → L (−0.36)	H → L + 1 (−0.47)				H → L + 2 (−0.33)
			H → L + 2 (0.21)						

a For completeness, the corresponding dominant orbital contributions for the benchmark annihilator TES-ADT are also reported. HOMO and LUMO are abbreviated as H and L, respectively. For a more comprehensive list, see Section S3.1 of the Supporting Information.

**Table 2:** Computed Electronic Properties of the Investigated Emitters at the B3LYP-D3/TD-B3LYP-D3 Level of Theory^a^

	tetracene	NH2T	NMe2T	NPh2OMeT	NO2T	DAT	TrAT1	TrAT2	TES-ADT
HOMO (S_0_)	–0.190	–0.163	–0.185	–0.171	–0.220	–0.212	–0.219	–0.223	–0.185
LUMO (S_0_)	–0.088	–0.087	–0.072	–0.093	–0.140	–0.119	–0.129	–0.131	–0.094
Δ*E*_HOMO–LUMOS0_	2.76	2.06	3.06	2.12	2.18	2.55	2.46	2.50	2.46
S_0_ → S_1_									
Δ*E*_vertS0→S1_	2.43	1.84	2.36	1.75	2.25	2.33	2.28	2.27	2.04
λ_absS0→S1_	511	674	526	709	552	532	544	546	608
*f*_S0→S1_	0.04	0.05	0.07	0.12	0.05	0.03	0.02	0.03	0.21
μ_S0→S1_	0.07	0.07	0.11	0.18	0.08	0.05	0.04	0.04	0.32
*k*_rad_^S_0_→S_1_^ (x10^4^)	7.31	3.69	16.2	17.6	8.65	3.01	1.83	2.32	93.4
Δ*E*_adiaS0‑S1_	2.25	1.58	1.90	1.61	1.91	2.15	2.09	2.08	1.93
Δ*E*_adiaZPVES0‑S1_	2.19	1.53	1.87	1.55	1.88	2.09	2.04	2.03	1.88
λ_absS0→S1EXP_	473^b^	–	521 (s), 496^c^	569^c^		502^b^	522^b^	594^b^	575^d^
S_0_ → T_1_									
Δ*E*_vertS0→T1_	1.16	0.82	1.11	1.01	1.08	1.19	1.21	1.17	0.96
λ_absS0→T1_	1065	1505	1116	1232	1143	1041	1023	1062	1290
λ_absS0→T1EXP_	925^e^								1148^d^
S_0_ → T_1_ (no TD)									
Δ*E*_vertS0→T1NOTD_	1.47	1.08	1.41	1.26	1.38	1.49	1.51	1.46	1.19
λ_absS0→T1NOTD_	845	1144	879	986	898	831	821	847	1039
Δ*E*_adiaS0‑T1NOTD_	1.21	0.82	1.06	1.02	1.10	1.21	1.21	1.17	1.00
Δ*E*_adiaZPVES0‑T1NOTD_	1.13	0.77	1.01	0.96	1.04	1.14	1.14	1.10	0.94
S_0_ → T_2_									
Δ*E*_vertS0→T2_	2.54	2.34	2.50	1.88	2.37	2.36	2.15	2.25	2.39
λ_absS0→T2_	487	530	496	659	523	525	578	552	520
S_1_ → S_0_									
Δ*E*_vertS1→S0_^′^	2.08	1.32	1.63	1.40	1.61	1.97	1.90	1.90	1.83
λ_fluoS1→S0_	596	938	762	885	771	629	652	654	678
*f*_S1→S0_	0.04	0.03	0.07	0.05	0.05	0.03	0.02	0.03	0.20
μ_S1→S0_	0.07	0.05	0.11	0.07	0.07	0.05	0.04	0.04	0.31
*k*_rad_^S_1_→S_0_^ (x10^4^)	4.28	0.63	5.33	1.52	2.23	1.65	0.953	1.21	62.8
λ_fluoS1→S0EXP_	–	–	–	615,^f^ 654^g^		533, ∼620 (s)^h^	576, ∼620 (s)^i^	571, ∼620 (s)^b^	580^d^

a HOMO and LUMO orbital energies of the ground state are given in hartree, while the corresponding HOMO–LUMO energy gaps (Δ*E*_HOMO–LUMOS0_) are reported in eV. For all transitions, the vertical excitation energies (Δ*E*_vert_), together with the corresponding absorption wavelengths (λ_abs_, nm), oscillator strengths (*f*, a.u.), transition dipole moments (μ, a.u.), and radiative decay rates (*k*_rad_, s^–1^) are reported. Adiabatic (Δ*E*_adia_) and zero-point vibrational energy–corrected adiabatic excitation energies (Δ*E*_adiaZPVE_) are also provided. For comparison, vertical (Δ*E*_vertS1→S0_^′^) de-excitation energies associated with the S_1_ → S_0_ transitions are listed, along with their corresponding emission wavelengths (λ_fluo_, nm). All energies are reported in eV unless otherwise specified. Experimental values from the literature are included when they are available.

b In THF, 2.7–3.2 × 10^–5^ M, λ_exc_ = 340 nm in the emission measurements, from ref 25.

c In CH_2_Cl_2_,, from ref 26.

d From refs 21,22 .

e From ref 49.

f In *n*-hexane, 1 × 10^–5^ M, λ_exc_ = 340 nm, from ref 26.

g In toluene, 1 × 10^–5^ M, λ_exc_ = 340 nm, from ref 26.

h In THF, 2.7–3.2 × 10^–5^ M,, λ_exc_ = 500 nm, from ref 25.

i In THF, 2.7–3.2 × 10^–5^ M, λ_exc_ = 530 nm, from ref 25.

Other considerations can be made based on the maps reported in Figures S2–S4. While these maps do not provide a direct measure of aromaticity, they offer qualitative insights into how electron density is redistributed within the π-system upon excitation. The conclusions drawn here are independently supported by ground-state NICS values, which show only minor variations upon functionalization and confirm that the aromatic framework of the tetracene core is retained. In all azaanthracene derivatives, the nitrogen centers act as regions of enhanced electron density, leading to marked deviations from unsubstituted tetracene in the local redistribution patterns, whereas the overall conjugated framework of the acene core remains intact. Consequently, the corresponding difference electron density maps show significant deviations from that of unsubstituted tetracene, while the overall aromatic character of the π-system remains largely preserved. The most pronounced variations relative to the parent tetracene occur around the central C–C bond of the aromatic core and the adjacent bonds, where a stronger redistribution of electron density is observed upon excitation. The persistence of an aromatic conjugation pattern is qualitatively reflected by the alternating regions of electron depletion and enrichment observed along the acene backbone in the difference-density maps for the S_0_ → T_1_ and S_0_ → S_1_ transitions. Importantly, this qualitative picture is corroborated by the NICS(1)_*zz* analysis reported in Table S2, which confirms that the intrinsic aromaticity distribution of the tetracene core is largely preserved in the azatetracenes.

In contrast, more pronounced local effects are observed for the 5,12-substitutes tetracenes at the carbon atoms directly bonded to the substituents. Specifically, for NH2T, as expected for an electron-donating group, the bonded carbon and the surrounding region are depleted of electron density (orange areas) compared to that of unsubstituted tetracene upon excitation. Conversely, in NO2T, the situation is reversed with an increase in electron density localized around the substituent-bound carbon, consistent with its electron-withdrawing character. The substituent in NPh2OMeT behaves differently than the other here considered: it consistently induces charge depletion on the substituent itself and a corresponding electron density enrichment on the tetracene backbone, irrespective of the transition considered. Interestingly, while for S_0_ → T_1_, the tetracene core still shows the alternating pattern of electron-depleted and electron-enriched bonds characteristic of aromatic systems, for S_0_ → S_1_ and S_0_ → T_2_, nearly all its bonds appear enriched in electron density, suggesting a substantial perturbation of the aromatic distribution. This unusual behavior likely reflects the extended conjugation of the diphenylamino group, combined with the strong electron-donating effect of the methoxy substituent, which together alter the balance between localized and delocalized orbital contributions. Accordingly, while NICS(1)_*zz* remains essentially unchanged in NPh2OMeT with respect to tetracene, indicating preservation of the global π-aromatic ring current, both NICS(0) and NICS(1) show a pronounced decrease for ring 2, i.e., the ring bearing the substituent, for which the values are the lowest within the series (see Table S2). This behavior points to a localized reduction of aromaticity induced by the substituent, which is not captured by the out-of-plane π-current descriptor alone.

Overall, the results indicate that functionalization does not disrupt the planarity of the tetracene core, implying that the tuning of excited-state properties is governed primarily by electronic rather than geometric effects. This is advantageous for TTA-upconversion because the preservation of planarity ensures favorable π–π stacking, whereas the substituent-dependent redistribution of electron density provides a handle to fine-tune intermolecular interactions. The case of NPh2OMeT is particularly illustrative: although the HOMO–LUMO alternation preserves the aromatic backbone (see Figure 1), its difference-density maps reveal a temporary redistribution of electron density upon excitation, highlighting how substituents can induce localized perturbations and modify the nature of the electronic excitations. This analysis is further confirmed by the NTO analysis (see Section S4 in the Supporting Information). Additionally, the NTO analysis confirms that the low-lying excited states considered here are predominantly single-excitation in nature, supporting the applicability of the chosen TD-DFT framework.

**Figure 1:**
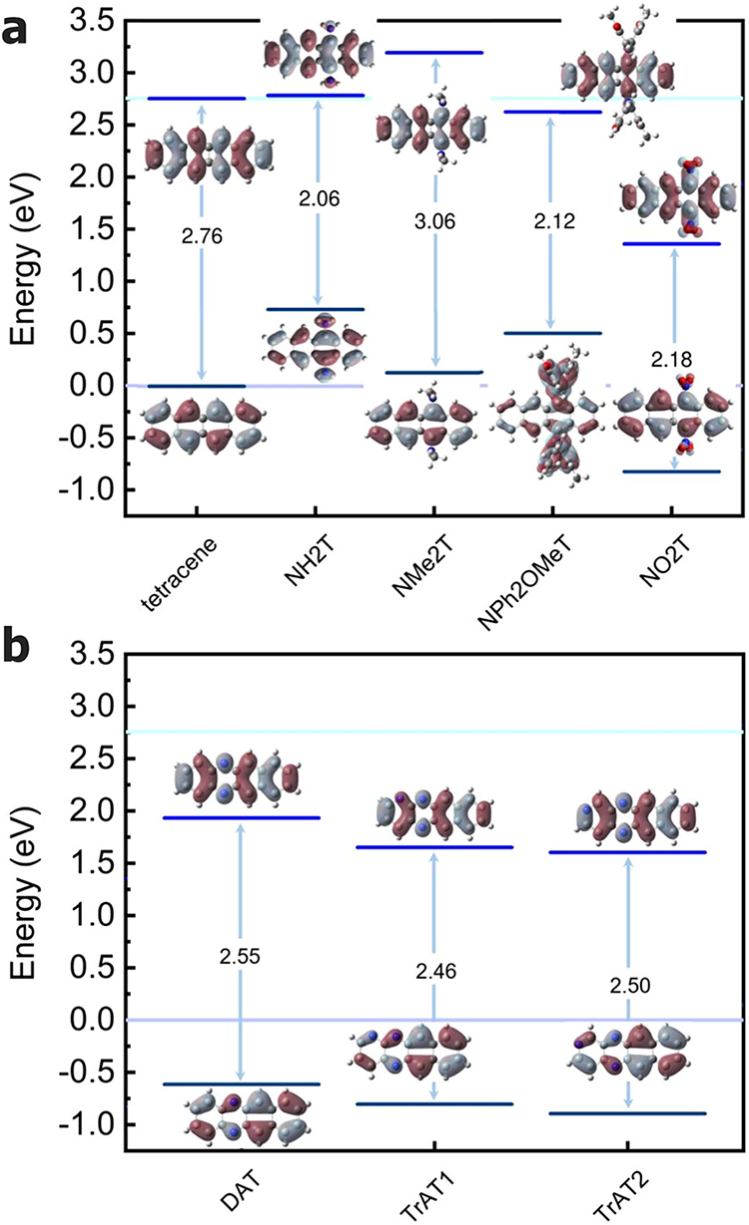
Kohn–Sham HOMO and LUMO and their energy levels in tetracenes. Values computed at the B3LYP-D3/def2-TZVP level for (a) tetracene and functionalized tetracene derivatives (Tetracene, NH2T, NMe2T, NPh2OMeT, and NO2T) and (b) nitrogen-doped tetracene cores (DAT, TrAT1, and TrAT2). HOMO in tetracenes was used as the zero of the energy. The Δ*E*_HOMO–LUMOS0_ values are reported in the plot in eV. Gray region: positive values; purple regions: negative values. Isovalues at 0.02 au. Light violet and light blue horizontal lines indicate the HOMO and LUMO energy of tetracene, respectively. Color code of the atoms: red (oxygen), blue (nitrogen), gray (carbon), and white (hydrogen).

The HOMO–LUMO energies (Δ*E*_HOMO–LUMOS0_) of the tetracenes are reported in Table 2 and Figure 1. They span a relatively broad range from 2.06 eV (NH2T) to 3.06 eV (NMe2T). Except for NMe2T, all tetracenes display smaller gaps than those of the parent compound (2.76 eV). For NH2T, NPh2OMeT, and NO2T, the gap is also reduced with respect to TES-ADT (2.46 eV, see Table 2). As shown in Figure 1, the functional groups affect both the HOMO and LUMO energies. Electron-donating groups, such as −NMe_2_ and −NH_2_, mainly raise the HOMO energy, whereas electron-withdrawing groups, such as −NO_2_ and pyridinic nitrogens, stabilize both frontier orbitals (see Figure 1). While affecting the energies, the spatial distribution of the HOMO and LUMO orbitals on the tetracene core remains unchanged across all derivatives, with only minor contributions from the substituents (see Figure 1). Also, for NPh2OMeT, where the HOMO shows a visible contribution from the diphenylamino substituent, its overall nodal structure and symmetry remain consistent with the tetracene-derived HOMO observed for the other derivatives, indicating an extension of conjugation rather than a change in orbital character.

The computed data for the S_0_ → S_1_, S_0_ → T_1_, and S_0_ → T_2_ transitions of the investigated emitters are summarized in Table 2 and illustrated in Figure 2.

**Figure 2:**
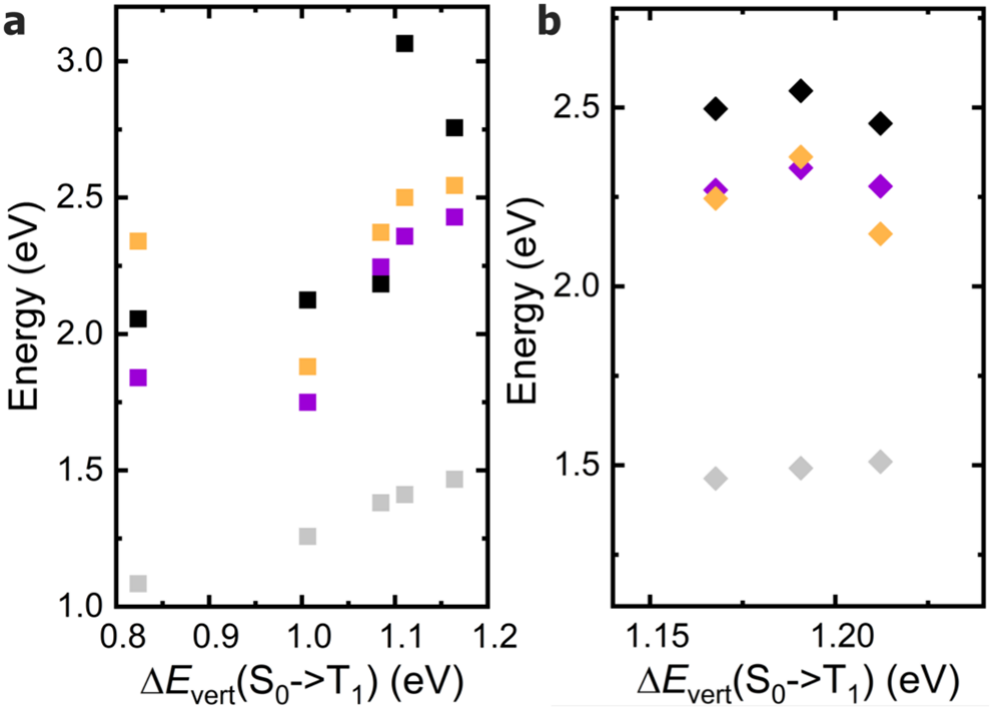
Correlation plot between the TD-B3LYP vertical S_0_ → T_1_ excitation energies (*x*-axis) and, on the *y*-axis, the HOMO–LUMO gaps (black), the TD-B3LYP vertical S_0_ → S_1_ excitation energies (violet), the TD-B3LYP vertical S_0_ → T_2_ (orange) excitation energies, and the vertical S_0_ → T_1_ excitation energies (gray) computed with standard B3LYP (*y*-axis). (a) substituted tetracenes, (b) azaarenes.

For the S_0_ → S_1_ transitions, the computed vertical excitation energies (Δ*E*_vertS0→S1_) are found between 1.75 eV (NPh2OMeT) and 2.43 eV (tetracene), translating into absorption maxima in the 511–709 nm range. The oscillator strengths (*f*) are generally low (0.02–0.12), typical of π–π* transitions in polyaromatic systems.^50^ Notably, the azatetracene derivatives exhibit the weakest transition dipole moments, even lower than tetracene, resulting in low radiative decay rates (*k*_rad_^S_0_→S_1_^ ≈ 1–3 × 10^4^ s^–1^). The state-of-the-art TES-ADT shows a markedly higher oscillator strength than all of the compounds (*f* = 0.21) and the largest computed *k*_rad_^S_0_→S_1_^ (9.34 × 10^5^ s^–1^), indicating a potentially more efficient fluorescence from the S_1_ state. The comparison between calculated and experimental λ_absS0→S1_ shows overall good agreement, with deviations within 20–30 nm for most compounds (i.e., errors lower than 0.2 eV, see Table 2). The only notable exception is NPh2OMeT, for which the computed transition is overestimated by nearly 150 nm (energy underestimated by 0.43 eV) relative to experiment. Inclusion of solvent effects (dichloromethane, as used in the experiments) does not reduce this discrepancy, but instead, it slightly increases the deviation (see Table S3). A possible explanation for this behavior is that TD-DFT is known to occasionally invert the relative oscillator strengths of close-lying excited states^51^ and that the experimentally observed band may correspond to S_2_ (computed at 619 nm) rather than S_1_ did not hold here. In fact, computing the excitation energies using the TD-CAM-B3LYP functional, which is often regarded as a benchmark for S_0_ → S_1_ transitions,^52^ for NPh2OMeT, the deviation with respect to experiment decreases substantially from +140 (TD-B3LYP) to −34 nm (TD-CAM-B3LYP, see Table S4). However, when considering the entire series, the average deviation using TD-CAM-B3LYP increases, indicating that the use of range-separated functionals does not systematically improve the predictions for this class of compounds. In particular, for TrAT2 the situation is reversed: TD-CAM-B3LYP underestimates λ_absS0→S1_ by −128 nm (+0.57 eV), whereas TD-B3LYP gives a much smaller deviation of −48 nm (+0.18 eV). For what concerns triplet energies, TD-CAM-B3LYP significantly underestimates all of them (up to 50%, see Table S4). Overall, these results suggest that the TD-B3LYP functional provides a balanced description of the excited states across the series, while TD-CAM-B3LYP may be required for specific systems with enhanced charge-transfer character, as in NPh2OMeT, characterized by a different nature of the transition than the other tetracenes as evident from the comparison of the maps in Figure S3. Additional tests involving TD-PBE0 including a CH_2_Cl_2_ polarizable continuum model were performed for NPh2OMeT following the indications from the literature.^40^ However, the resulting λ_absS0→S1_ remains comparably overestimated with respect to the experiment (694 versus 569 nm).

Triplet energies follow the same trends upon substitution as singlet energies. They were evaluated using two methods: B3LYP and TD-B3LYP. The S_0_ → T_1_ transitions computed using B3LYP (see Table 2) lie between 1.08 (NH2T) and 1.51 eV (TrAT1), with λ_absS0→T1_ spanning 821–1144 nm. The corresponding values computed at the TD-B3LYP level are systematically lower by about 20–30 nm, spanning from 0.82 (NH2T) to 1.21 eV (TrAT1). The two available experimental values lie between the B3LYP-D3 and TD-B3LYP-D3 results, with a deviation smaller than 0.18 eV (see Table 2). This indicates that the two computational approaches likely bracket the true excitation energy. Actually, the triplet energies calculated with the two methods show a linear correlation across all of the molecules considered (see gray symbols in Figure 2). Such a level of agreement lends confidence to the overall reliability of the adopted computational protocol, at least for this class of emitters. Additionally, because of the significantly lower computational cost of B3LYP than TD-B3LYP, this suggest its potential adoption in large screenings to evaluate the triplet energies. Among the tetracenes, NH2T is predicted to have a Δ*E*_vertS0→T1_ red-shifted with respect to TES-ADT between 215 (TD-B3LYP-D3) and 105 nm (B3LYP-D3), which would correspond to a triplet energy comprised between 1253 and 1363 nm, i.e., in the telecom band.

Importantly for TTA-UC applications, the singlet–triplet gaps (Δ*E*_vertS0→S1_ – Δ*E*_vertS0→T1_) are consistently in the range 0.6–1.0 eV for all the compounds, i.e., large enough to have a significant gain in energy in the TTA-UC process, when a suitable sensitizer is employed (see also below the discussion on the singlet energy loss). Efficient TTA-UC also requires (i) T_2_ to be substantially higher in energy than T_1_ and (ii) S_1_ to lie below T_2_, preventing the system from decaying to T_2_ rather than relaxing to S_1_ in the final step of the UP process. Criterion (i) is satisfied for all compounds studied, with T_2_ approximately twice the energy of T_1_ (1.88–2.48 eV). Criterion (ii) holds for all systems except TrAT1 and TrAT2, where T_2_ is located below S_1_, potentially opening nonradiative decay pathways and then reducing the overall upconversion yield (see Section S3.1). Thus, their suitability as TTA-UC emitters appears significantly reduced compared to the other candidates considered here. While the relative energetic positioning of S_1_, T_1_, and T_2_ states suggests that spin-forbidden decay channels may be possible in principle for TrAT1 and TrAT2, a quantitative evaluation of spin–orbit coupling effects and associated nonradiative rates requires dedicated multistate calculations and is therefore left for future work.

To gain further insights into the internal consistency of the computed excitation energies and to identify possible correlations between different descriptors, Δ*E*_vertS0→T1_ were plotted against the HOMO–LUMO gap and the other vertical excitation energies reported in Table 2 (see Figure 2a,b). No clear correlation emerges between the HOMO–LUMO gap (black symbols) and the vertical excitation energies, indicating that simple frontier orbital energetics are not sufficient to rationalize the excited-state behavior of these systems. In contrast, a near-linear correlation is observed between the vertical S_0_ → S_1_ (violet) and S_0_ → T_2_ (orange) excitation energies for tetracene, NH2T, NMe2T, and NO2T, which lie approximately on a common straight line (Figure 2a). These results indicate that when the two transitions have comparable character across a given class of compounds, a direct relationship between their transition energies may emerge. Regarding azatetracenes, since only three such heteroatom-containing species are included, any general conclusion must be drawn with caution. Nonetheless, the results in Figure 2b and in Table 2 suggest that heteroatom substitution perturbs the electronic structure less than the introduction of external functional groups, at least in terms of excitation energies.

A graphical approach for evaluating the potential of a given emitter for effective photon upconversion was reported by Wang et al.^53^ In this diagram, the *y*-axis represents the triplet energy loss, reflecting the thermodynamic favorability of the triplet channel being closed, defined as

$$E_{\mathrm{loss}}^{\mathrm{TTA},T}=2\Delta E_{S0\to T1}-\Delta E_{S0\to T2}$$

where the transition energies are approximated by their vertical excitation energies. In particular, more negative values correspond to higher theoretical quantum efficiency for the emitter since they indicate that the triplet–triplet annihilation (TTA) decay leads preferentially to the singlet product.

On the *x*-axis, another key energetic parameter is shown: the singlet energy loss, defined as

$$E_{\mathrm{loss}}^{\mathrm{TTA},S}=2\Delta E_{S0\to T1}-\Delta E_{S0\to S1}$$

where, as for the *y*-axis, the transition energies are approximated by their vertical excitation energies. This value represents how thermodynamically favorable the TTA-UC process is. The more positive this energy difference, the more favorable the singlet formation pathway becomes. However, if this value becomes too large, then the energy gain in the emitted photon is reduced, limiting the practical advantage of photon upconversion. Wang et al. suggested that this energy loss should ideally not exceed that observed for pyrene.^53^ Rubrene is known to undergo nearly isoenergetic TTA-UC with a closed triplet channel. To account for systematic computational errors, we recentered the diagram by setting both energy loss values computed for rubrene at this level of approximation to zero. The ideal region for an efficient emitter is indicated by the light blue area in Figure 3. It is important to stress that this framework does not provide a quantitative estimate of the upconversion quantum yield but rather identifies energetic regions that are favorable or unfavorable for TTA-UC. This energy loss plot is therefore proposed here as a convenient and intuitive visualization tool to be routinely included in the computational studies of TTA-UC annihilators. Although it is grounded in the thermodynamic constraints of the process, it provides an immediate qualitative assessment and enables a first screening of the most promising candidate emitters. In particular, this plot serves as a qualitative descriptor to identify annihilators for which the formation of the S_1_ state is thermodynamically favored over population of higher-lying triplet states (*E*_loss_^TTA,T^ > 0). From a fundamental perspective, the theoretical upper limit of TTA-UC is constrained by spin statistics: the entangled pair [T_1_ T_1_], can populate singlet (1/9), triplet (3/9), or quintet (5/9) manifolds, leading to a nominal maximum of 5.6% UC efficiency relative to absorbed photons. In practice, this limit can be partially relaxed if decay from the quintet and triplet-pair states is suppressed, particularly when T_2_ lies significantly higher in energy than that of 2T_1_. In the idealized limit where triplet and quintet pair states are efficiently recycled and do not act as loss channels, the intrinsic two-photon-to-one-photon nature of TTA sets an upper bound of 50% for the upconversion quantum yield relative to absorbed photons.

**Figure 3:**
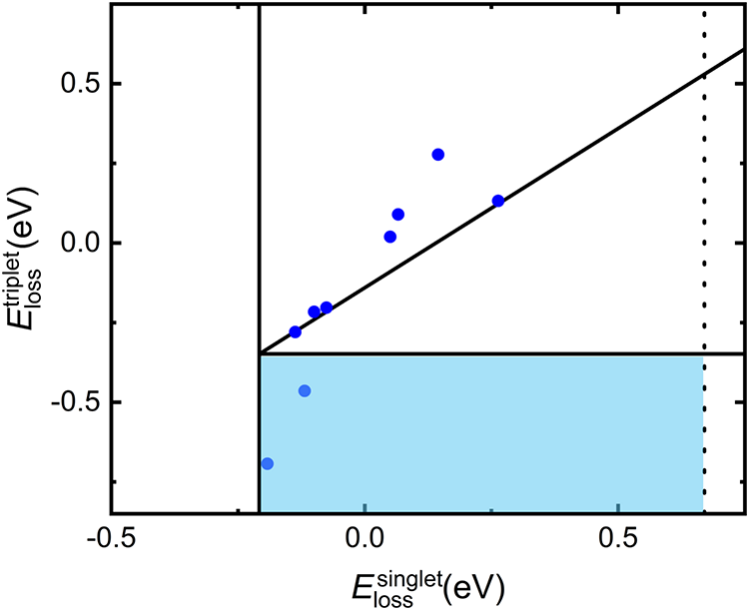
Energy loss diagram for the TTA-UC emitters considered in this work. The solid vertical and horizontal lines correspond to the singlet and triplet energy loss of rubrene, respectively, taken as reference (zero) based on previous experimental results.^53^ The dashed vertical line indicates the singlet energy loss of pyrene, above which no thermodynamic advantage is expected for TTA-UC. The blue area represents the ideal region for an efficient emitter.^53^ The straight line marking the equivalence of the singlet and triplet energy losses, corrected for the systematic errors, is also reported for comparison (*y* = *x* – 0.14). All values were obtained at the TD-B3LYP-D3/def2-TZVP level of theory.

Only two compounds in the diagram fall within this blue-shaded region: TES-ADT and NH2T. The latter is therefore not only capable of absorbing radiation in a spectral range that has not yet been explored for TTA-UC, but also appears promising from a thermodynamic standpoint. As an additional and indirect consistency check, it is worth noting that TES-ADT has been experimentally reported to exhibit high TTA-UC efficiencies.^22^ While this does not constitute a validation of the present descriptor, it supports its usefulness as a qualitative screening tool for identifying promising annihilators.

### 3.2 Comparison of S_0_ → S_1_ and S_1_ → S_0_ Transitions

While vertical S_0_ → S_1_ excitation energies are often employed as proxies for emission properties, our data allow for a direct comparison between computed S_0_ → S_1_ and S_1_ → S_0_ energies. As summarized in Table 2 and visualized in Figure 4, the experimental values reported in the literature (red points in the plot) indicate that the vertical de-excitation energies (λ_fluoS1→S0EXP_) are systematically lower than the corresponding excitation energies (λ_absS0→S1EXP_). This (obvious) result is consistent with the expected stabilization of the S_1_ state upon geometric relaxation. The review of the data further allows quantification of this effect in tetracenes, with an energy difference of 0.09 eV for TrAT2 to 0.28 and 0.46 eV for NPh2OMeT and DAT, respectively. This difference is far from negligible and suggests that absorption-derived singlet energies should be employed with caution as proxies for emission energies, particularly in tetracene-based systems. Exceptions exist, such as the state-of-the-art TES-ADT, showing a remarkable coincidence between the two values, with a Stokes shift of only 5 nm,^22^ which is also significantly lower than that of related compounds (e.g., ∼30 nm for F_2_-TES-ADT).^21^ These results suggest that although the higher computational cost associated with excited-state geometry optimization makes it unsuitable for large-scale screening, the energy difference between the S_0_ → S_1_ and S_1_ → S_0_ transitions should be explicitly quantified for the most relevant systems. This highlights the importance of including excited-state geometry optimization when the aim is to predict emission wavelengths quantitatively. The comparison between the computed and experimental emission wavelengths shows that the method accurately reproduces the experimental data only for the azatetracene derivatives (within 9–34 nm), while larger discrepancies are observed for the other systems. In particular, the emission wavelength is overestimated by approximately 100 nm for the reference compound TES-ADT (energy error of −0.31 eV) and by more than 200 nm for NPh2OMeT (−0.5 eV).

**Figure 4:**
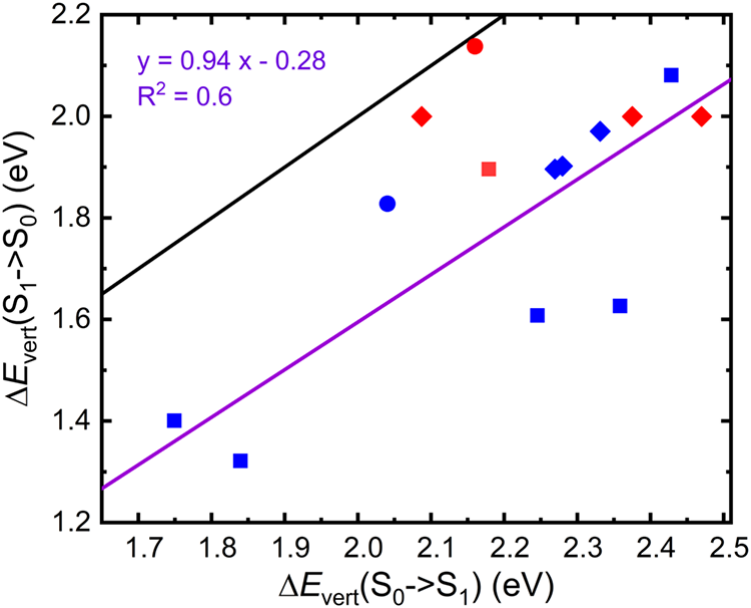
Comparison between the vertical S_0_ → S_1_ and S_1_ → S_0_ transition energies computed at the TD-B3LYP/def2-TZVP level (blue symbols). The degree of deviation from the diagonal (black line) reflects the extent of excited-state structural relaxation with smaller differences indicating more rigid molecular frameworks and potentially reduced vibrational losses upon emission. The violet straight line corresponds to linear fits of the computed data. The experimental data are reported in red. Azarenes (diamonds), TES-ADT (circles), and others (squares).

As shown in Figure 4, the computed data points (blue) cluster along a line that is nearly parallel to the diagonal (linear fit: *y* = 0.94*x* – 0.28), indicating that the computed S_0_ → S_1_ vertical excitation energies can be considered as a systematic underestimation of the corresponding S_1_ → S_0_ vertical emission energies by an almost constant offset. Despite the limited number of points, the trend suggests that the excited-state relaxation and the associated reorganization energies introduce a non-negligible Stokes shift, consistent with the experimental observations.

This analysis leads to two important implications. First, this confirms that absorption and emission energies cannot be used interchangeably when quantitatively estimating the energetic gain of the TTA-UC process. Second, the nearly linear correlation across the series suggests that, for screening purposes, computing the S_0_ → S_1_ excitation energies may still serve as a reliable first-order descriptor of relative performance, owing to their substantially lower computational cost. Accurate S_1_ → S_0_ emission energies, requiring excited-state geometry optimizations, should instead be reserved for the most promising candidates for which a quantitative assessment of the upconversion energetics is necessary. A larger data set would be needed to assess whether this linear dependence is maintained across different emitter families or depends on the specific molecular framework.

A comparison between the radiative decay rates estimated using the standard S_0_ → S_1_ approximation (*k*_rad_^S_0_→S_1_^, see Table 2), and those explicitly computed for the S_1_ → S_0_ emission (*k*_rad_^S_1_→S_0_^) reveals systematic differences across the series. In all cases, *k*_rad_^S_1_→S_0_^ is lower than the corresponding *k*_rad_^S_0_→S_1_^, with reductions ranging from a factor of ∼1.5 for tetracene and TES-ADT up to more than 1 order of magnitude for strongly substituted derivatives such as NH2T and NPh2OMeT. This trend reflects the combined effect of excited-state structural relaxation and reduced transition dipole moments, highlighting the limitations of the ground-state-based approximation for quantitative predictions of emission properties. The reduction of *k*_rad_^S_1_→S_0_^ relative to the corresponding S_0_ → S_1_ estimates arises from two concurrent effects: (i) the stabilization of the excited state upon structural relaxation, leading to a lower emission energy (Stokes shift), and (ii) a decrease of the transition electric dipole moment. Since the radiative decay rate scales with both the cube of the transition energy and the square of the transition dipole moment, even moderate changes in these quantities result in substantial variations of *k*_rad_.

Among the systems investigated, NH2T emerged as the most promising candidate in our screening. The computed fluorescence spectrum for NH2T indicates an emission energy corresponding to 938 nm, compared to an absorption maximum at 674 nm (see also comparison between solid and dashed blue lines in Figure 5 and the spectra in Figures S5 and S11). Although the computational method tends to overestimate emission wavelengths compared to the experiments (by more than 200 nm for structurally related compound NPh2OMeP), the predicted emission would remain outside the visible range. This outcome is fully consistent with the intrinsic energetic constraints of the TTA process: two photons at 1505 nm (triplet energy for NH2T at the TD-B3LYP level) yield a theoretical energy equivalent to 750 nm, which is insufficient to drive NIR-to-visible upconversion. Consequently, these emitters cannot, by design, operate as NIR-to-visible annihilators for telecom-band excitation. For applications targeting this spectral region, a new generation of TTA-UC emitters will therefore be required for NIR-to-NIR upconversion. Achieving visible upconversion with this class of emitters would then require their introduction into a cascade system capable of extending the energy range. In this context, tetracene could represent a reasonable next step in a cascade scheme, as suggested by the energetic data summarized in Table 2 and their overlap with the calculated NH2T emission.

**Figure 5:**
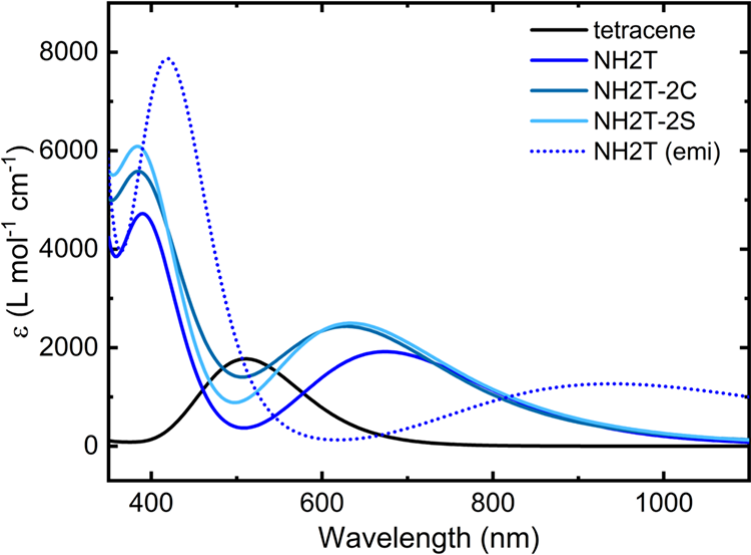
Spectroscopic features of the most red-shifted TTA-UC chromophore. TD-B3LYP-D3/def2-TZVP computed UV–Vis absorption spectra including the first 100 excitations of NH2T in its monomeric form and in two dimeric configurations, NH2T-2C and NH2T-2S. For comparison, the spectrum of tetracene is also reported. The emission spectrum for monomeric NH2T is shown as a dashed line. The full computed range is available in Figure S11 of the Supporting Information.

### 3.3 Dimers

Since TTA-UC relies on the interaction of two triplet-excited chromophores, it is important to extend the analysis from monomers to their corresponding dimers^54,55^ for the most promising candidate, NH2T, and the parent compound. The optimized geometries for the dimers in S_0_ of tetracene and NH2T are reported in Figures 6a–d and S9. Optimization attempts for tetracene dimers starting from different input geometries all converged to two possible final structures: a skewed-parallel geometry (2S) and a coaxial-parallel geometry (2C). In both cases, the molecules lie on parallel, noncoincident planes; however, in 2S, their molecular axes are not parallel, whereas in 2C, they are. As tetracene is concerned, 2S was consistently found to be more stable than the 2C, although the energy difference is modest: only about 5 kJ·mol^–1^. Given this small energy gap, both geometries were considered in the analysis, as it is likely that both coexist in the devices. For NH2T, the presence of polar functional groups stabilizes the dimer through intermolecular interactions, leading all optimized structures to adopt the 2C configuration, although they exhibit slight variations in monomer–monomer distance and overall molecular planarity (see Figures 6, S9 and Table S5). In the following, I refer to the NH2T dimers using the 2S and 2C labels to indicate the input structures from which they were generated, rather than the final optimized geometry, which correspond to the 2C type in both cases, although they are nonetheless different. Notably, in the NH2T-2S structure, the amino groups on the same tetracene are oriented in the same way with respect to the tetracene plane, while in NH2T-2C, they have the hydrogen atoms oriented in an opposite way: this results in the NH2T-2S having a slightly higher stability than NH2T-2C by approximately 6 kJ·mol^–1^. Despite their similar relative energies, these slightly different molecular orientations are expected to affect orbital overlap in different ways and, consequently, the efficiency of the TTA-UC process. For this reason, all four structures were considered in the discussion.

**Figure 6:**
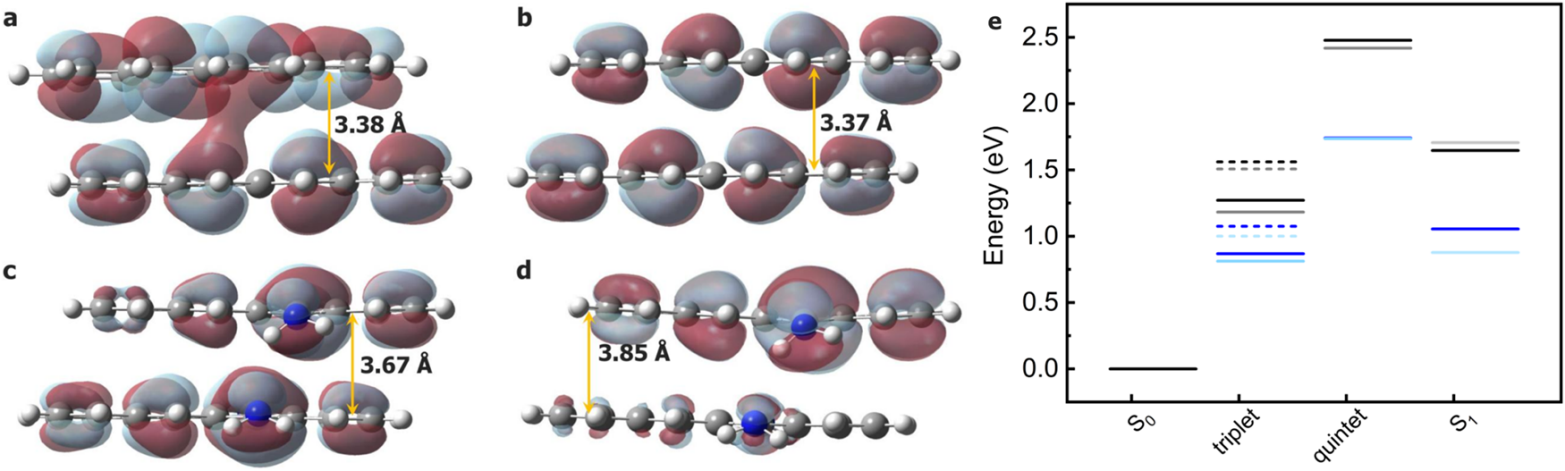
Molecular stacking structure and orbital overlap in the dimers. Optimized geometries for (a) tetracene-2S, (b) tetracene-2C, (c) NH2T-2S, and (d) NH2T-2C were obtained at the B3LYP-D3/def2-TZVP level of theory in the ground state and in the gas phase. The HOMO orbitals are reported. Gray regions: positive values, purple regions: negative values. Color code: gray (carbon), white (hydrogen), blue (nitrogen). The maximum interplanar separation between the two monomers is indicated in the figure for each dimer. The corresponding top view of the dimers is reported in Figure S9. (e) Spin ladder for the tetracene-2S (black), tetracene-2C (gray), NH2T-2S (blue), and NH2T-2C (light blue) dimers (adiabatic energies). For the triplet, the energies related to electronic configuration A (solid lines) and configuration B (dashed line) are both reported.

The dimer formation energies are found to lie between −66 and −95 kJ mol^–1^ (Δ*E*_dimerS0_^c^, see Table 3). For tetracene, as previously discussed, the 2S configuration is the most stable, with a formation energy of −70.5 kJ·mol^–1^, compared to 94.8 kJ·mol^–1^ for NH2T-2S, which is more stable due to the presence of hydrogen-bonding interactions between the amino groups on the two monomers. The presence of these functional groups also leads to an increase in the intermonomer distance for NH2T dimers compared to the unsubstituted tetracene dimers, from 3.37 Å to 3.67 and 3.85 Å, otherwise counterintuitive because of the smaller Δ*E*_dimerS0_^c^. On the geometrical point of view, it can be noted that dimerization decreases the planarity of the tetracene core, with NH2T showing the most marked deviation from planarity, particularly in the NH2T-2C configuration (see Figure 6d and Tables S1 and S5).

**Table 3:** Computed Structural and Energetic Parameters for the Optimized Dimers in Different Electronic States^a^

	T-2S	T-2C	NH2T-2S	NH2T-2C
Δ*E*_dimerS0_	–74.3	–69.1	–101.8	–95.9
Δ*E*_dimerS0_^c^	–70.5	–65.7	–94.8	–89.1
Δ*H*_dimerS0_^c^	–64.3	–59.5	–87.5	–81.7
Δ*G*_dimerS0_^c^	–10.1	–6.0	–28.1	–23.7
Δ*E*_HOMO–LUMO_	2.7	2.6	2.0	1.8
Δ*E*_vertS0→S1_	2.19	2.14	1.63	1.31
λ_absS0→S1_	566	578	761	946
*f*_S0→S1_	0.00	0.02	0.01	0.00
μ_S0→S1_	0.00	0.03	0.02	0.00
Δ*E*_vertS0→T1_	1.23	1.16	0.85	0.82
λ_absS0→T1_	1011	1071	1466	1507
Δ*E*_adiaS0→T1NOTD‑A_	1.56	1.51	1.08	1.00
Δ*E*_adiaS0→T1NOTD‑B_	1.27	1.18	0.87	0.81
Δ*E*_adiaS0→S1_	1.65	1.71	1.05	0.88
Δ*E*_adiaS0→Q1_	2.48	2.42	1.74	1.74

a Reported quantities include the electronic (Δ*E*_dimerS0_) and BSSE-corrected stabilization energies (Δ*E*_dimerS0_^*c*^), enthalpies (Δ*H*_dimerS0_^*c*^), and Gibbs free energies (Δ*G*_dimerS0_^*c*^, in kJ mol^–1^), and selected electronic properties: HOMO–LUMO energy gap (Δ*E*_HOMO‑LUMO_, in eV), vertical excitation energies (Δ*E*_vert_ in eV) and corresponding absorption wavelengths (λ_abs_ in nm), oscillator strengths (*f*_abs_ in a.u.), and transition dipole moments (μ_abs_ in a.u.) for the S_0_ → S_1_ and S_0_ → T_1_ transitions. Adiabatic energy gaps for the relevant excited states (Δ*E*_adia_, in eV) are also listed. All data refer to computed electronic properties of the investigated emitters at the B3LYP-D3/TD-B3LYP-D3 level of theory.

The HOMO is strongly affected by both the relative orientation and the distance between the monomers. As shown in Figure 6 (side view) and Figure S9 (top view), tetracene-2S is the only complex in which the HOMO exhibits significant orbital overlap between the two monomers, indicating a relative monomer arrangement that may favor intermolecular electronic coupling relevant to the TTA-UC process. Interestingly, a similar overlap is also present in the LUMO of the quintet (T_1_T_1_) state (see Figure S10). In contrast, the amino substituents in NH2T stabilize coaxial configurations with a much lower intermonomer orbital overlap. Synthetic strategies capable of stabilizing 2S-type configurations even in amino-substituted systems, for instance, through incorporation into extended frameworks, could therefore represent a promising route to improve upconversion efficiency. A direct comparison between NH2T-2S and NH2T-2C shows that increasing the monomer separation leads to a more localized HOMO, with a reduced electron density shared between the two monomers. This reduced delocalization diminishes the electronic coupling between the chromophores, which is potentially detrimental for TTA-UC, as it lowers the probability of triplet–triplet coupling. While a quantitative assessment of TTA rates and upconversion efficiencies would require an explicit kinetic treatment beyond the scope of the present work, the orbital overlap analysis provides a qualitative descriptor of relative intermolecular coupling across different stacking motifs. Since the 2C dimer is only about 6 kJ·mol^–1^ less stable than 2S, it is expected to be significantly populated at room temperature. Using a Boltzmann distribution at 298 K, we estimate that approximately 10% of the dimers should populate the 2C configuration, which is thus expected to contribute to a reduction in the effective intermolecular coupling relevant for the TTA-UC performance relative to a hypothetical system containing exclusively 2S dimers.

The UV–Vis absorption spectra of the dimers in their ground state (S_0_) are reported in the 350–1200 nm range in Figure 5 and S11. The corresponding excitation energies for the S_0_ → S_1_ and S_0_ → T_1_ transitions are summarized in Table 3. Compared to their monomeric counterparts, the dimers exhibit markedly different spectral features in the 200–350 nm region, characterized by a decrease in the number of observable bands and a reduction in the molar extinction coefficients. In contrast, in the 350–1600 nm range, the monomeric and dimeric spectra are very similar, although a blue shift of the bands is observed as expected for H-aggregates.^56^ By contrast, when one looks at the energy values of the S_0_ → S_1_ transition, the formation of the dimer is always accompanied by a red shift. In particular, it is red-shifted from 511 nm to 566 (2S) and 578 nm (2C) in the tetracene dimer, and from 674 nm to 761 (2S) and 946 nm (2C) in the NH2T dimer. Analysis of the difference-density maps for these excitations (Figure S13) indicates that the S_0_ → S_1_ transition is happening on each monomer in a similar way as in the isolated form for unfunctionalized tetracene, while for NH2T-2C and NH2T-2S, the excitation has a completely different character and involves the displacement of the electron density from one monomer to the other. This justifies the small shift upon dimer formation in tetracene with respect to the larger ones observed for NH2T. Conversely, the S_0_ → T_1_ transition in the dimers shows only minor energy changes relative to the monomers, shifting from 1065 to 1011 nm (2S) and 1071 nm (2C) in tetracene, and from 1505 to 1466 nm (2S) and 1507 nm (2C) in NH2T. The corresponding difference-density maps confirm that the excitation is very similar to that observed for the isolated monomeric unit, consistent with the minimal perturbation of the transition energy (Figures S2 and S12, for the monomers and the dimers, respectively).

Geometry optimizations were carried out for the T_1_, S_1_, and Q_1_ states, in order to reconstruct the spin ladder in the dimers (see Figure 6e). This approach enabled us to examine how molecular geometries and spin–state interaction energies change across different electronic configurations, thus providing a more accurate description of the potential energy surface. Another objective of this work was to quantify, from a computational perspective, the extent to which the molecular geometry is affected by changes in the electronic state. This is particularly important because of the commonly used approximation of performing single-point calculations of excited states on the ground-state geometry, a practice often necessary to reduce computational cost. Geometry optimization of the dimers in the triplet state were conducted considering two distinct electronic configurations (Table S5): (i) a symmetric configuration (A), in which the spin density is equally distributed over the two monomers, and (ii) an asymmetric configuration (B), where one monomer adopts a triplet state and the other remains in a singlet state. In both cases, spin contamination was negligible with ⟨S^2^⟩ = 2.003, very close to the ideal value of 2.0 for a pure triplet. Geometrically, in both cases the monomers are slightly closer than in S_0_ (*d* values in Table S5), with no significant impact on the molecular planarity (*d*_avg_ values in Table S5). For configuration A, the triplet dimer lies approximately 1.5 eV (tetracene) and 1.0 eV (NH2T) above the singlet ground state (solid lines in Figure 6e), values that are higher than the monomer triplet energies for tetracene and NH2T (1.21 and 0.82 eV, respectively). For configuration B, the triplet energies are significantly lower than those for configuration A but comparable to the Δ*E*_adia_ in the monomer (1.27–1.18 eV for tetracene and 0.8 eV for NH2T dimers; dashed lines in Figure 6e). This behavior is expected since configuration A corresponds to an entirely different electronic arrangement than the monomeric triplet, whereas configuration B essentially represents a dimer in which one monomer is in the S_0_ state and the other in the T_1_ state. Consequently, the adiabatic energies of the dimers and the monomers are expected to be similar. This suggests that configuration B can be regarded as a realistic model for a [T_1_···S_0_] precursor in the TTA process.

Among the investigated states, Q_1_ is the most relevant, as it represents the electronic structure closest to that expected after triplet–triplet collisions: ^1^[T_1_···T_1_]. Although this open-shell singlet state (that is, the antiferromagnetically coupled counterpart of Q_1_) is slightly lower in energy, Q_1_ provides the most accessible approximation of the ^1^[T_1_···T_1_] state and also of the entangled triplet-pair state ^1^[T_1_ T_1_] at the DFT level. It should be emphasized that the Q_1_ state is used here as a static DFT-level proxy to extract structural information about the triplet-pair dimer. A quantitative description of triplet–triplet entanglement, nonadiabatic couplings, and possible excimer formation would require multireference electronic-structure methods and lies beyond the scope of the present study but represents a natural direction for future studies. Spin population analysis (Table S5) confirms that both monomers retain their triplet character in this configuration. While electronically distinct from the entangled triplet-pair state ^1^[T_1_ T_1_], the core of the TTA-UC process,^21^ the Q_1_ model provides valuable structural insights. Notably, the comparison of key structural parameters between S_0_ and Q_1_ shows that the geometry of the dimer remains essentially unchanged upon triplet excitation. This minimal structural rearrangement is particularly significant for TTA design: minimal reorganization energy implies reduced nonradiative losses and potentially higher upconversion efficiency. On the energetic point of view, the spin ladder indicates for the dimers that the energetic stability follows the expected order singlet > triplet > quintet (see Figure 6e and Table 3). In particular, Q_1_ is less stable than S_0_ by ∼2.45 and 1.74 eV in tetracene and NH2T, respectively.

## 4 Conclusions

Photon upconversion through triplet–triplet annihilation enables the conversion of low-energy photons into higher-energy emission,^57–59^ but no TTA-UC system operates within the 1250–1675 nm region, which is particularly relevant for telecommunications and quantum information technologies.

In this work, we combine targeted molecular modification, including heteroatom incorporation, with quantum-chemical modeling to computationally identify 5,12-bis­(*N*,*N*-diamino)­tetracene as a promising emitter candidate for TTA-UC in this spectral range. In particular, NH2T is predicted to exhibit an S_0_ → T_1_ excitation energy lower than that of state-of-the-art annihilators, falling within the telecom band. In addition, other 5,12-diamino-tetracene derivatives are predicted to be viable candidates for experimental testing as TTA emitters for NIR-to-visible upconversion, albeit at energies higher than those of NH2T.

Detailed electronic-structure analyses were performed on both monomeric and dimeric forms, exploring multiple electronic configurations to gain insights into the potential energy landscape of the triplet-pair complex. A key finding is that the optimized dimer geometries exhibit only a minor structural rearrangement upon electronic excitation. This observation provides qualitative support for the common approximation of evaluating higher-level electronic-structure methods at ground-state geometries in future studies.^60^

Furthermore, we propose the previously reported energy-loss plot as a convenient and intuitive visualization tool enabling rapid qualitative assessment of candidate TTA-UC annihilators within computational screening workflows.^53^

Our results also indicate that dimer arrangements in which the fused-ring cores lie on parallel planes but with non-parallel molecular axes exhibit enhanced orbital overlap between monomer units. Such geometries are expected to facilitate more efficient triplet–triplet energy transfer and annihilation, thus guiding the design of improved TTA-UC emitters.

The amino substituents markedly reduce the triplet energy, opening an unprecedented window for TTA emitters in the telecom band. The modest structural complexity of NH2T, together with existing synthetic precedents for closely related diamino-tetracenes, suggests that experimental realization may be feasible using adaptations of established synthetic approaches. This compelling combination calls for experimental confirmation, with future computational efforts aimed at rationalizing the remarkable efficacy of amino groups, guiding the design of next-generation TTA systems.

## Supplementary Material





## Notes

Computed electronic absorption and fluorescence spectra, comprehensive list of the orbitals contribution to the main electronic transitions, excitation energies computed in dichloromethane, excitation energies computed at the TD-CAM-B3LYP level, and supplementary electronic and geometric properties of the monomers and the dimers (PDF)
Cartesian coordinates of computational models and ASCII version of the electronic spectra (ZIP)

## Abbreviations

TTA-UC: triplet–triplet annihilation upconversion
KS-DFT: Kohn–Sham density functional theory
TD: time dependent
NICS: nucleus-independent chemical shift
NTO: natural transition orbital
