# Supplementary material for: Breaking the 1250 nm Barrier: A Computational Approach to Light Upconversion via Triplet–Triplet Annihilation in the Silica Telecom Band
Source: ACS Appl Energy Mater. 2026 Feb 12;9(4):2398–411. doi: 10.1021/acsaem.5c04014 (PMC12934551; doi:10.1021/acsaem.5c04014)
Supplement: Supplementary file 1 [file ae5c04014_si_001.pdf]

Supporting Information

Breaking the 1250 nm Barrier: A Computational Approach to Light Upconversion via Triplet-Triplet Annihilation in the Silica Telecom Band

Jenny G. Vitillo [a,*]

[a]Department of Science and High Technology and INSTM, Università degli Studi dell'Insubria, Via Valleggio 9, 22100 Como, Italy.

*e-mail: jg.vitillo@gmail.com

Table of Contents

S1. Supplementary geometrical data for all the monomers in the ground and excited states 3

S2. Supplementary data for estimating changes in monomer aromaticity 4

S3. Supplementary theoretical UV-Vis absorption and fluorescence spectra and data for the monomers 9

S3.1. TD-B3LYP-D3: Excitation energies and orbital contribution in the absorption spectra computed for S_0 13

Tetracene 13

NH2T 13

NMe2T 14

NPh2OMeT 14

NO2T 15

DAT 15

TrAT1 16

TrAT2 16

S3.2. TD-B3LYP-D3: Dexcitation energies and orbital contribution in the fluorescence spectra computed for S_1 17

Tetracene 17

NH2T 18

NMe2T 19

NPh2OMeT 19

NO2T 20

DAT 21

TrAT1 21

TrAT2 22

S4. Supplementary theoretical UV-Vis absorption data for the monomers: natural transition orbitals analysis for the $S_0 \rightarrow T_1$, $S_0 \rightarrow S_1$, and $S_0 \rightarrow T_2$ transitions 23

S5. Supplementary theoretical UV-Vis absorption data in dichloromethane 28

S6. Supplementary theoretical UV-Vis absorption data at the TD-CAM-B3LYP-D3 level of theory 29

S7. Supplementary data on the dimers 30

S7.1. TD-B3LYP-D3: Excitation energies and orbital contribution in the absorption spectra computed for S_0 36

NH2T dimers 2S 36

NH2T dimers 2C 37

Tetracene dimer 2S 38

Tetracene dimer 2C 39

S7.2. TD-B3LYP-D3: Dexcitation energies and orbital contribution in the fluorescence spectra computed for S_1 39

NH2T dimers 2S 39

NH2T dimers 2C 40

Tetracene dimer 2S 40

Tetracene dimer 2C 41

S8. Supplementary energetic data for all the systems 42

S9. Coordinates of all the optimized structures 45

References 45

S1. Supplementary geometrical data for all the monomers in the ground and excited states

The main geometrical and electronic properties of tetracenes are reported in Table S1.

Table S1. Geometrical and electronic properties of tetracenes in ground and excited states. Out-of-plane displacements of the atoms above (R_{above}) and below (R_{below}) the mean plane defined by the tetracene core are reported in Å as the average absolute distance (d_{avg}) of the atoms from this plane, providing a measure of the distortion induced by functionalization. Atomic charges (q) and spin densities (ρ) on the tetracene core (C and N atoms only) are given for all emitters (in a.u.). Values obtained at the (TD)B3LYP-D3BJ/def2-TZVP level of theory.

	T	NH2T	NMe2T	NPh2OMeT	NO2T	DAT	TrAT1	TrAT2
**S_0**								
d_{avg}	0.000	0.025	0.000	0.011	0.009	0.000	0.000	0.000
R_{above}	0.000	0.555	2.079	5.771	1.027	0.000	0.000	0.000
R_{below}	0.000	0.553	2.077	6.570	1.014	0.000	0.000	0.000
$\rho_{Hirshfeld}$	0.00	0.00	0.00	0.00	0.00	0.00	0.00	0.00
$q_{Hirshfeld}$	-0.48	-0.45	-0.38	-0.34	-0.20	-0.45	-0.42	-0.42
q_{CM5}	-1.16	-0.89	-0.81	-0.77	-0.62	-1.03	-0.96	-0.96
**S_1**								
d_{avg}	0.000	0.025	0.026	0.031	0.027	0.000	0.000	0.000
R_{above}	0.000	0.493	1.780	6.176	0.802	0.000	0.000	0.000
R_{below}	0.000	0.493	1.792	6.793	0.775	0.000	0.000	0.000
$\rho_{Hirshfeld}$	0.00	0.00	0.00	0.00	0.00	0.00	0.00	0.00
$q_{Hirshfeld}$	-0.48	-0.47	-0.44	-0.30	-0.17	-0.45	-0.42	-0.42
q_{CM5}	-1.16	-0.90	-0.87	-0.74	-0.59	-1.03	-0.95	-0.96
**T_1 (NO TD)**								
d_{avg}	0.000	0.025	0.105	0.014	0.018	0.000	0.000	0.000
R_{above}	0.000	0.580	1.877	5.460	0.818	0.000	0.000	0.000
R_{below}	0.000	0.580	1.876	6.425	0.981	0.000	0.000	0.000
$\rho_{Hirshfeld}$	1.90	1.64	1.66	1.61	1.75	1.93	1.93	1.94
$q_{Hirshfeld}$	-0.47	-0.51	-0.48	-0.43	-0.15	-0.45	-0.43	-0.43
q_{CM5}	-1.16	-0.94	-0.91	-0.86	-0.57	-1.03	-0.96	-0.97

S2. Supplementary data for estimating changes in monomer aromaticity

Aromaticity variations in the investigated monomers were estimated using nucleus-independent chemical shift (NICS) indices. In particular, NICS(1)_zz was adopted, as it is widely regarded in the literature as the most reliable descriptor for assessing π-aromaticity.[1, 2] For the NICS calculations, molecular geometries were generally retained with the same orientation used in the electronic-structure calculations, as the tetracene core lies approximately in the XY plane for all derivatives, with only minor deviations arising from functional-group-induced distortions.

An exception is NPh2OMeT, which exhibits a more pronounced nonplanarity due to the bulky substituents. For this molecule only, the structure was reoriented prior to the NICS calculations to ensure a well-defined out-of-plane direction relative to the tetracene core. The Cartesian coordinates of the reoriented structure used for the NICS analysis are reported below:

C	9.73706209	0.00262373	0.08298256
C	9.73724950	-1.42283412	0.08052945
C	8.56543656	-2.11435551	0.07735818
C	7.31182726	-1.43119624	0.07745681
C	7.31148618	0.01086677	0.08021823
C	8.56518559	0.69394200	0.08233205
C	6.10238906	0.68829851	0.07382057
C	4.87258204	0.01201770	0.07682921
C	4.87287116	-1.43324756	0.07424913
C	6.10279777	-2.10889380	0.06845973
C	3.64000745	-2.12653492	0.03618595
C	2.42876015	-1.43296458	0.00000000
C	2.42870809	0.01091393	0.00109997
C	3.63960144	0.70468899	0.03999103
C	1.17236157	0.69009665	0.00408731
C	0.00000000	0.00000000	0.00000000
C	0.00000000	-1.42178400	0.00000000
C	1.17225875	-2.11184738	0.00350868
H	10.68133182	-1.95224982	0.08259221
H	8.56146747	-3.19734423	0.07813818
H	6.10851202	-3.18875158	0.05765526
H	1.16902571	1.76924843	0.01115751
H	-0.94151932	0.53367782	0.00423211
H	-0.94155927	-1.95547239	0.00452357
H	1.16848017	-3.19101035	0.00980839
H	6.10764523	1.76812461	0.06779832
H	8.56117237	1.77704775	0.08651481
H	10.68112655	0.53214931	0.08656423
N	3.64376293	-3.54792536	0.03009486
N	3.64105232	2.12657603	0.03720817
C	3.24988075	-4.22619332	-1.14261271
C	2.55725104	-5.43393967	-1.09286142
C	3.52049211	-3.66914628	-2.39771837
C	2.16003412	-6.08353495	-2.25683477
H	2.32314581	-5.87664307	-0.13479590
C	3.10974124	-4.29929316	-3.55664375
H	4.05357954	-2.73053847	-2.45794404

C	2.43073194	-5.51674423	-3.49971941
H	1.62846509	-7.01984917	-2.17546923
H	3.31781257	-3.86536312	-4.52550252
C	4.03516930	-4.23073872	1.20079825
C	3.78075400	-3.67924031	2.45316306
C	4.73194972	-5.44497843	1.14077402
C	4.20821146	-4.30788775	3.61882743
H	3.25003696	-2.73986604	2.52140768
C	5.14056318	-6.08335325	2.29492050
H	4.95501363	-5.88547935	0.17914575
C	4.88645916	-5.52046101	3.54751731
H	3.99641513	-3.84249600	4.56976982
H	5.67934341	-7.02037394	2.24716602
C	3.26573974	2.80522766	-1.14160510
C	2.57303629	4.01343100	-1.10158788
C	3.55138596	2.24710403	-2.39282731
C	2.18997337	4.66182356	-2.27075018
H	2.32789056	4.45712151	-0.14676176
C	3.15508178	2.87643189	-3.55745684
H	4.08442869	1.30807990	-2.44578537
C	2.47575728	4.09373845	-3.50996198
H	1.65686941	5.59797968	-2.19698777
H	3.37459747	2.44126652	-4.52321333
C	4.03542532	2.80756109	1.20779996
C	4.72778447	4.01583419	1.15646458
C	3.77202649	2.24933690	2.46392731
C	5.13289412	4.66266820	2.31899227
H	4.95601006	4.46057695	0.19798563
C	4.18985515	2.87773901	3.62158754
H	3.24018493	1.31018197	2.52560158
C	4.86953291	4.09409701	3.56292298
H	5.66462418	5.59889981	2.23599356
H	3.98758307	2.44197079	4.59091510
O	5.33969165	-6.22308927	4.62569034
O	2.13777198	4.64370286	-4.71226272
O	2.07823469	-6.06728347	-4.69751779
O	5.23010160	4.64276779	4.75954206
C	5.93857397	5.86713172	4.74302563
H	6.89295240	5.77055674	4.21597798
H	6.12682566	6.12132516	5.78345151
H	5.35074831	6.66404972	4.27671056
C	1.37645328	-7.29566217	-4.68397164
H	1.96590604	-8.08858003	-4.21267145
H	1.19664257	-7.55223556	-5.72534919
H	0.41814659	-7.20371713	-4.16332261
C	5.11454307	-5.68131421	5.91342542
H	5.59696407	-4.70538114	6.02653023
H	5.55390200	-6.38380401	6.61773362
H	4.04535109	-5.57924352	6.12346340
C	1.43175982	5.86957399	-4.70789543
H	0.46793617	5.77492143	-4.19816012
H	1.26300408	6.12465298	-5.75151172
H	2.01302255	6.66500749	-4.23077739

In Table S2, NICS(0), NICS(1), and NICS(1)_zz values are reported for all tetracene derivatives. Two definitions of NICS(1)_zz are provided: (i) NICS(1)_zz_a, obtained directly from the ZZ component of the magnetic shielding tensor; (ii) NICS(1)_zz_b, obtained as the largest eigenvalue of the shielding tensor, which corresponds to the out-of-plane component and is independent of the molecular orientation. NICS(1)_zz_a and NICS(1)_zz_b provided similar trends. Unless

otherwise stated, NICS(1)_zz values discussed in the main text refer to NICS(1)_zz_b. The aromatic rings were numbered as shown in Figure S1.

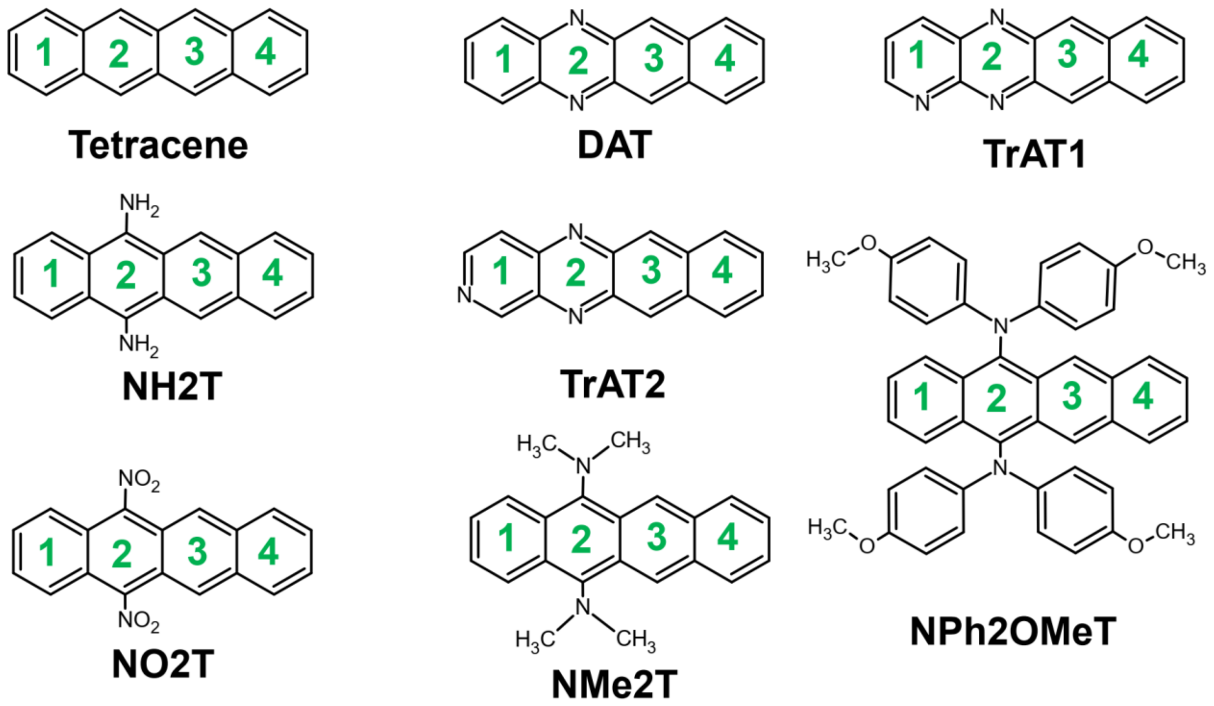

Figure S1. ChemDraw representations of tetracene and its functionalized derivatives investigated in this work. The four fused rings of the tetracene core are labeled in green (rings 1–4) to define the positions used for the NICS analysis.

Table S2. Nucleus-independent chemical shift (NICS, in ppm) values computed for the tetracene core of all investigated derivatives in their ground state. NICS(0), NICS(1), and NICS(1)_zz values are reported for each of the four fused rings, numbered as shown in Figure S1. NICS(1)_zz values are provided both as the ZZ component of the magnetic shielding tensor (NICS(1)_zz(a)) and as the largest eigenvalue of the tensor (NICS(1)_zz(b)). Values obtained at the (TD)B3LYP-D3BJ/def2-TZVP level of theory.

	tetracene	NH2T	NMe2T	NPh2OMeT	NO2T	DAT	TrAT1	TrAT2
**NICS(0)**								
ring 1	6.31	6.23	6.67	6.61	6.74	5.78	4.92	4.71
ring 2	11.17	11.14	11.82	10.07	13.89	8.85	9.16	9.01
ring 3	11.17	10.76	11.32	11.18	11.40	11.02	10.92	10.83
ring 4	6.31	5.95	6.08	6.27	6.32	6.32	6.28	6.22
**NICS(1)**								
ring 1	8.67	8.19	8.78	8.44	8.88	8.56	8.55	8.58
ring 2	12.81	11.61	12.57	10.66	12.34	12.59	12.66	12.72
ring 3	12.81	12.22	12.79	12.71	12.64	12.74	12.68	12.65
ring 4	8.67	8.50	8.53	8.93	8.90	8.74	8.74	8.70
**NICS(1)_zz_a**								
ring 1	23.93	22.19	23.07	22.86	24.11	23.97	23.11	23.67
ring 2	35.36	29.74	32.16	29.72	32.24	33.78	33.85	33.85
ring 3	35.36	33.24	34.22	33.23	34.62	35.43	35.47	35.20
ring 4	23.93	23.10	23.29	23.25	24.29	24.29	24.33	24.15
**NICS(1)_zz_b**								
ring 1	24.30	22.65	23.50	23.26	24.39	24.07	23.40	23.78
ring 2	35.38	29.77	32.18	29.76	32.25	33.83	33.93	33.92
ring 3	35.38	33.30	34.25	33.23	34.67	35.43	35.48	35.21
ring 4	24.30	23.55	23.71	23.57	24.74	24.62	24.68	24.50

For pristine tetracene, the NICS values reproduce the expected trend of higher aromatic character for the inner rings (rings 2 and 3) compared to the terminal rings (rings 1 and 4), consistent with the well-known aromaticity distribution in linearly fused acenes.[1] This pattern is preserved across all substituted derivatives, indicating that functionalization does not qualitatively alter the aromatic framework of the tetracene core. Electron-donating substituents $–NH_2$ and $–N(PhOMe)_2$ induce a moderate reduction of NICS(1)_zz values, particularly for the inner rings, consistent with partial delocalization of electron density away from the acene core. In contrast, $–NMe_2$ and the electron-withdrawing $–NO_2$ substituent slightly increases the NICS(1)_zz values (although always lower than in tetracene), suggesting a marginal enhancement of ring current strength within the core. For heteroatom-substituted derivatives (DAT, TrAT1, and TrAT2), the NICS(1)_zz values remain comparable to those of tetracene, with only minor variations between rings. This indicates that nitrogen incorporation within the acene backbone preserves the overall aromatic character while subtly redistributing it across the fused rings. Overall, the NICS analysis confirms that the modifications explored in this work tune the electronic structure primarily through frontier orbital energetics, while leaving the intrinsic aromaticity pattern of the tetracene scaffold mostly intact.

S3. Supplementary theoretical UV-Vis absorption and fluorescence spectra and data for the monomers

Difference of the total electronic density of the T_1, S_1, and T_2 state and the ground state are found in Figure S2, Figure S3, and Figure S4 respectively.

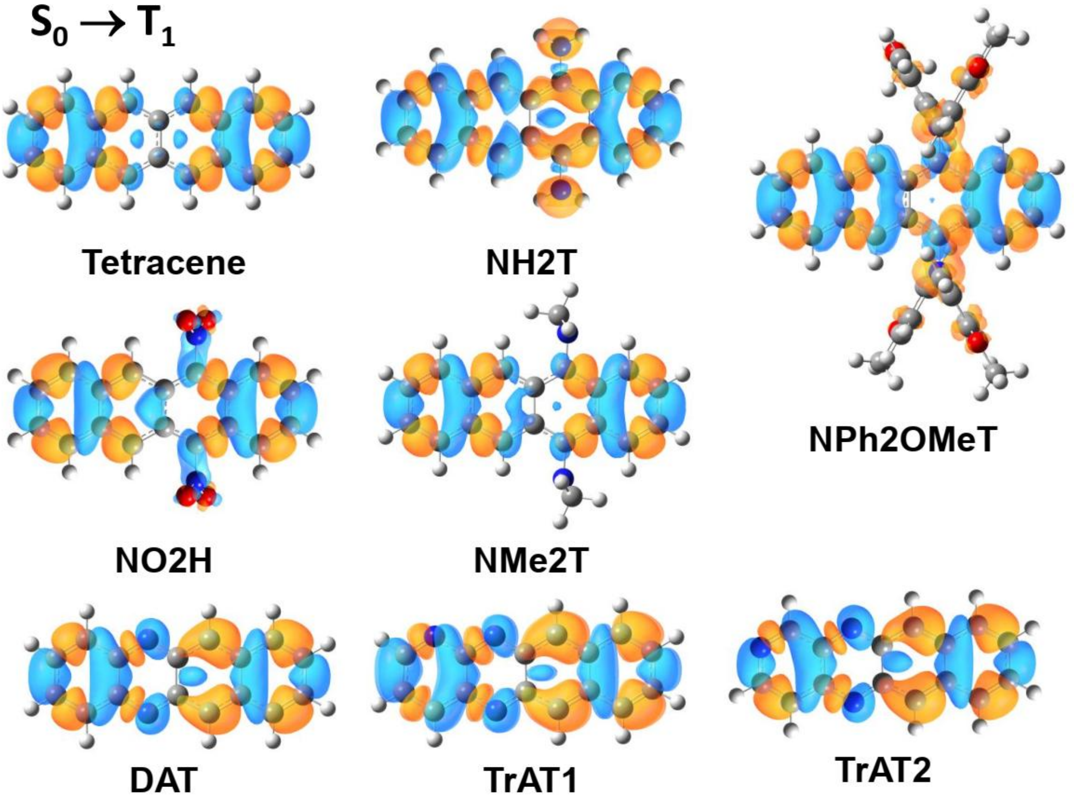

Figure S2. Difference of the total electronic density of the first excited triplet state and the ground state in all the tetracene derivatives included in this study ($S_0 \rightarrow T_1$ transition). Values computed at the TD-B3LYP-D3/def2-TZVP. Blue regions: positive values, corresponding to an increase of the total electronic density in the excited state with respect to S_0. Orange regions: negative values. Isosurface at 0.0006 a.u. Color code of the atoms: red (oxygen), blue (nitrogen), grey (carbon), white (hydrogen).

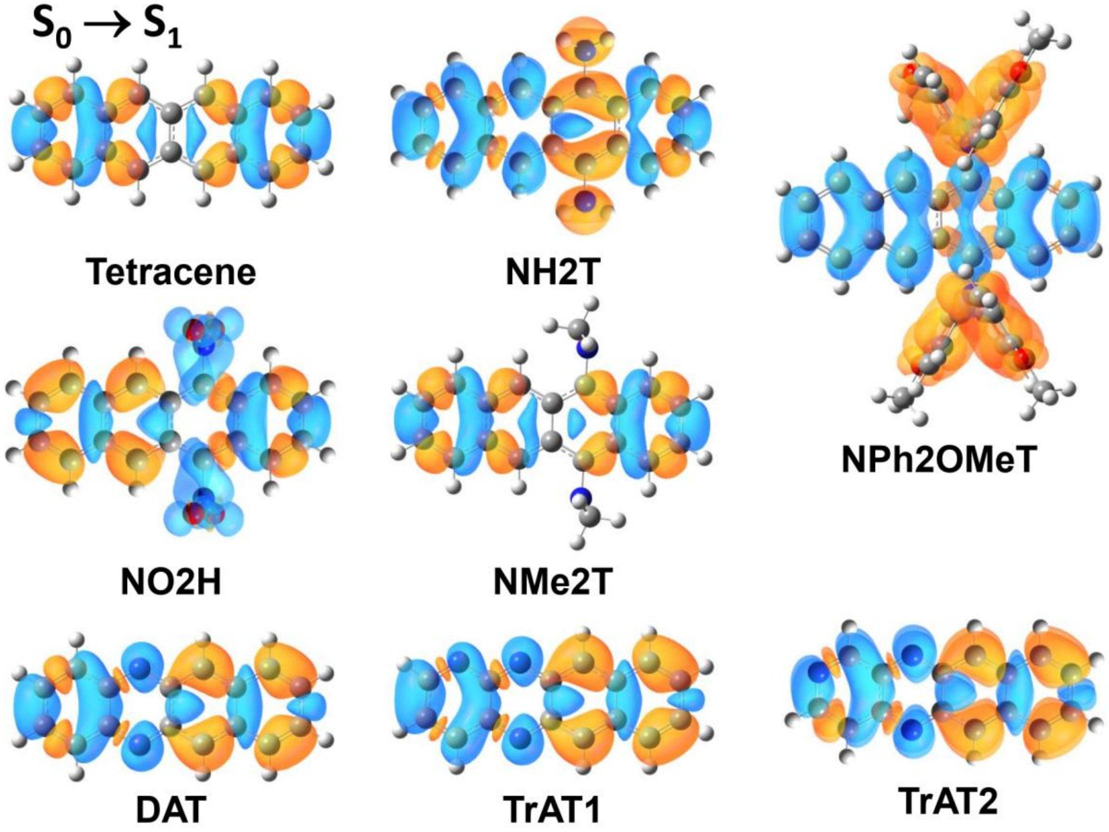

Figure S3. Difference of the total electronic density of the first excited singlet state and the ground state in all the organic molecules included in this study ($S_0 \rightarrow S_1$ transition). Values computed at the TD-B3LYP-D3/def2-TZVP. Color codes as in Figure S2.

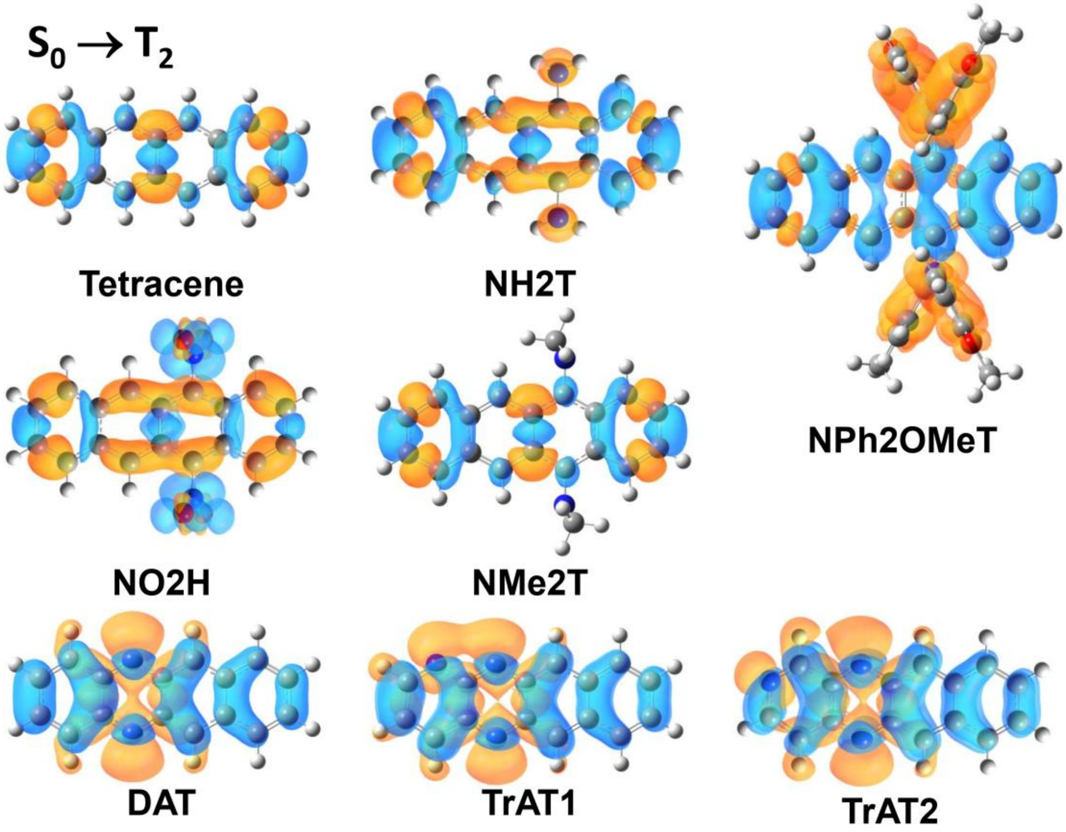

Figure S4. Difference of the total electronic density of the second excited triplet state and the ground state in all the organic molecules included in this study ($S_0 \rightarrow T_2$ transition). Values computed at the TD-B3LYP-D3/def2-TZVP. Color codes as in Figure S2.

The absorption and fluorescence spectra exhibit highly similar profiles, as anticipated from their structural similarity. Minor variations between S_0 and S_1 in the positions of functional groups relative to the acene core are observed, yet they influence solely the peak widths.

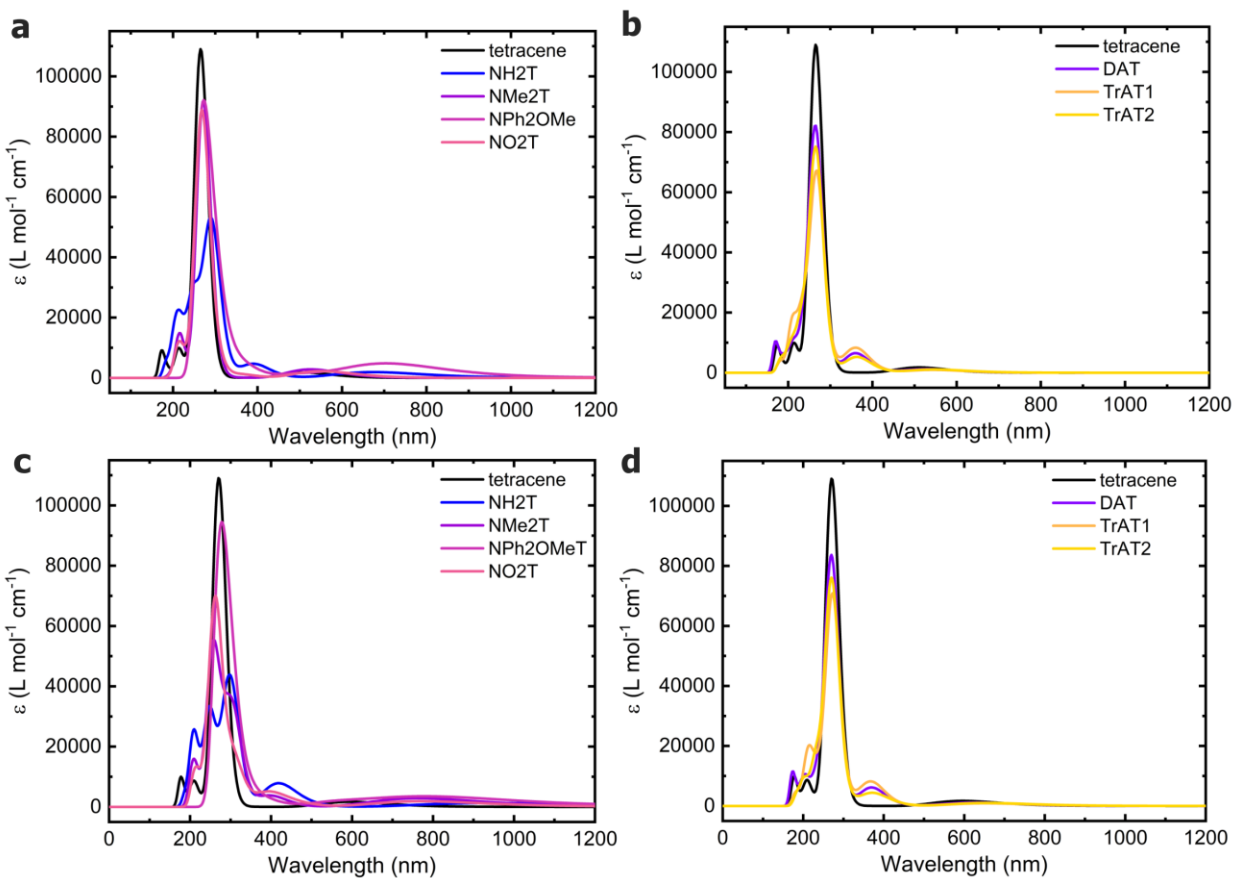

Figure S5. Electronic absorption and fluorescence spectra as computed at the TD-B3LYP-D3/def2-TZVP including the first 100 (de)excitations. **a,b.** absorption spectra. **c,d.** fluorescence spectra.

S3.1. TD-B3LYP-D3: Excitation energies and orbital contribution in the absorption spectra computed for S_0

Tetracene

60 HOMO, 61 LUMO

```
Excited State   1:  3.000-A'     1.1643 eV 1064.88 nm  f=0.0000  <S**2>=2.000
     59A -> 62A       -0.12270
     60A -> 61A        0.71186
     59B -> 62B        0.12270
     60B -> 61B       -0.71186
     60A <- 61A        0.17701
     60B <- 61B       -0.17701
 This state for optimization and/or second-order correction.
 Total Energy, E(TD-HF/TD-DFT) =  -693.439199951
 Copying the excited state density for this state as the 1-particle RhoCI density.

 Excited State   2:  1.000-A'     2.4284 eV  510.55 nm  f=0.0438  <S**2>=0.000
     60A -> 61A        0.70594
     60B -> 61B        0.70594

 Excited State   3:  3.000-A'     2.5447 eV  487.22 nm  f=0.0000  <S**2>=2.000
     59A -> 61A        0.52678
     60A -> 62A       -0.44661
     59B -> 61B       -0.52678
     60B -> 62B        0.44661

 Excited State   4:  3.000-A'     3.1694 eV  391.19 nm  f=0.0000  <S**2>=2.000
     58A -> 61A        0.64106
     60A -> 63A        0.28110
     58B -> 61B       -0.64106
     60B -> 63B       -0.28110
```

[…]

NH2T

68 HOMO, 69 LUMO

```
Excited State   1:  3.000-A      0.8238 eV 1505.10 nm  f=0.0000  <S**2>=2.000
     68A -> 69A       -0.71952
     68B -> 69B        0.71952
     68A <- 69A       -0.19624
     68B <- 69B        0.19624
 This state for optimization and/or second-order correction.
 Total Energy, E(TD-HF/TD-DFT) =  -804.213545933
 Copying the excited state density for this state as the 1-particle RhoCI density.

 Excited State   2:  1.000-A      1.8397 eV  673.95 nm  f=0.0474  <S**2>=0.000
     68A -> 69A        0.70713
     68B -> 69B        0.70713

 Excited State   3:  3.000-A      2.3406 eV  529.72 nm  f=0.0000  <S**2>=2.000
     67A -> 69A        0.48585
     67A -> 71A       -0.11525
     68A -> 71A       -0.47725
     67B -> 69B       -0.48585
     67B -> 71B        0.11525
     68B -> 71B        0.47725

 Excited State   4:  3.000-A      2.6552 eV  466.94 nm  f=0.0000  <S**2>=2.000
     68A -> 70A       -0.70072
```

```
     68B -> 70B         0.70072
```

[…]

NMe2T

84 HOMO, 85 LUMO

```
Excited State   1:  3.000-A      1.1106 eV 1116.36 nm  f=0.0000  <S**2>=2.000
     81A -> 86A       -0.10339
     84A -> 85A       -0.71410
     81B -> 86B        0.10339
     84B -> 85B        0.71410
     84A <- 85A       -0.18349
     84B <- 85B        0.18349
 This state for optimization and/or second-order correction.
 Total Energy, E(TD-HF/TD-DFT) =  -961.485973756
 Copying the excited state density for this state as the 1-particle RhoCI density.

 Excited State   2:  1.000-A      2.3584 eV  525.70 nm  f=0.0699  <S**2>=0.000
     84A -> 85A        0.70636
     84B -> 85B        0.70636

 Excited State   3:  3.000-A      2.5004 eV  495.87 nm  f=0.0000  <S**2>=2.000
     81A -> 85A        0.54083
     84A -> 86A        0.37598
     84A -> 87A        0.20662
     81B -> 85B       -0.54083
     84B -> 86B       -0.37598
     84B -> 87B       -0.20662

 Excited State   4:  3.000-A      2.6610 eV  465.93 nm  f=0.0000  <S**2>=2.000
     82A -> 85A        0.19496
     83A -> 85A       -0.67540
     82B -> 85B       -0.19496
     83B -> 85B        0.67540
```

[…]

NPh2OMeT

180 HOMO, 181 LUMO

```
Excited State   1:  3.000-A      1.0063 eV 1232.02 nm  f=0.0000  <S**2>=2.000
    178A -> 181A       0.38151
    180A -> 181A       0.60448
    178B -> 181B      -0.38151
    180B -> 181B      -0.60448
    178A <- 181A       0.12614
    180A <- 181A       0.12254
    178B <- 181B      -0.12614
    180B <- 181B      -0.12254
 This state for optimization and/or second-order correction.
 Total Energy, E(TD-HF/TD-DFT) =  -2187.13547050
 Copying the excited state density for this state as the 1-particle RhoCI density.

 Excited State   2:  1.000-A      1.7493 eV  708.75 nm  f=0.1166  <S**2>=0.000
    178A -> 181A      -0.11923
    180A -> 181A       0.69621
    178B -> 181B      -0.11923
    180B -> 181B       0.69621
```

```
 Excited State   3:  3.000-A      1.8801 eV  659.46 nm  f=0.0000  <S**2>=2.000
    178A -> 181A       0.57627
    179A -> 181A      -0.10573
    180A -> 181A      -0.36148
    178B -> 181B      -0.57627
    179B -> 181B       0.10573
    180B -> 181B       0.36148

 Excited State   4:  3.000-A      1.9199 eV  645.78 nm  f=0.0000  <S**2>=2.000
    179A -> 181A       0.69551
    179B -> 181B      -0.69551
```

[…]

NO2T

82 HOMO, 83 LUMO

```
Excited State   1:  3.000-A      1.0849 eV 1142.84 nm  f=0.0000  <S**2>=2.000
     81A -> 87A        0.11362
     82A -> 83A        0.70427
     82A -> 84A       -0.10772
     81B -> 87B       -0.11362
     82B -> 83B       -0.70427
     82B -> 84B        0.10772
     82A <- 83A        0.17692
     82B <- 83B       -0.17692
 This state for optimization and/or second-order correction.
 Total Energy, E(TD-HF/TD-DFT) =  -1102.61149678
 Copying the excited state density for this state as the 1-particle RhoCI density.

 Excited State   2:  1.000-A      2.2454 eV  552.18 nm  f=0.0546  <S**2>=0.000
     82A -> 83A        0.69371
     82A -> 84A        0.12808
     82B -> 83B        0.69371
     82B -> 84B        0.12808

 Excited State   3:  3.000-A      2.3727 eV  522.55 nm  f=0.0000  <S**2>=2.000
     81A -> 83A        0.41538
     82A -> 84A       -0.47195
     82A -> 87A        0.27282
     81B -> 83B       -0.41538
     82B -> 84B        0.47195
     82B -> 87B       -0.27282

 Excited State   4:  3.000-A      2.5337 eV  489.34 nm  f=0.0000  <S**2>=2.000
     79A -> 85A       -0.10351
     82A -> 85A       -0.67686
     79B -> 85B        0.10351
     82B -> 85B        0.67686
```

[…]

DAT

60 HOMO, 61 LUMO

```
Excited State   1:  3.000-A'     1.1907 eV 1041.26 nm  f=0.0000  <S**2>=2.000
     57A -> 62A       -0.10828
     60A -> 61A        0.70440
```

```
     57B -> 62B        0.10828
     60B -> 61B       -0.70440
     60A <- 61A        0.16305
     60B <- 61B       -0.16305
 This state for optimization and/or second-order correction.
 Total Energy, E(TD-HF/TD-DFT) =  -725.521430163
 Copying the excited state density for this state as the 1-particle RhoCI density.

 Excited State   2:  1.000-A'     2.3311 eV  531.86 nm  f=0.0292  <S**2>=0.000
     60A -> 61A        0.70422
     60B -> 61B        0.70422

 Excited State   3:  3.000-A"     2.3619 eV  524.93 nm  f=0.0000  <S**2>=2.000
     59A -> 61A       -0.68964
     59B -> 61B        0.68964

 Excited State   4:  3.000-A'     2.5059 eV  494.76 nm  f=0.0000  <S**2>=2.000
     57A -> 61A        0.60344
     60A -> 62A       -0.33562
     57B -> 61B       -0.60344
     60B -> 62B        0.33562
```

[…]

TrAT1

60 HOMO, 61 LUMO

```
Excited State   1:  3.000-A'     1.2122 eV 1022.80 nm  f=0.0000  <S**2>=2.000
     57A -> 61A        0.10009
     60A -> 61A       -0.69771
     60A -> 62A       -0.11596
     57B -> 61B       -0.10009
     60B -> 61B        0.69771
     60B -> 62B        0.11596
     60A <- 61A       -0.15224
     60B <- 61B        0.15224
 This state for optimization and/or second-order correction.
 Total Energy, E(TD-HF/TD-DFT) =  -741.560104331
 Copying the excited state density for this state as the 1-particle RhoCI density.

 Excited State   2:  3.000-A"     2.1468 eV  577.53 nm  f=0.0000  <S**2>=2.000
     59A -> 61A        0.68838
     59B -> 61B       -0.68838

 Excited State   3:  1.000-A'     2.2797 eV  543.87 nm  f=0.0236  <S**2>=0.000
     60A -> 61A        0.70382
     60B -> 61B        0.70382

 Excited State   4:  1.000-A"     2.4816 eV  499.61 nm  f=0.0001  <S**2>=0.000
     59A -> 61A        0.70251
     59B -> 61B        0.70251
```

[…]

TrAT2

60 HOMO, 61 LUMO

```
Excited State   1:  3.000-A'     1.1677 eV 1061.77 nm  f=0.0000  <S**2>=2.000
```

```
     60A -> 61A       -0.70344
     60B -> 61B        0.70344
     60A <- 61A       -0.16161
     60B <- 61B        0.16161
 This state for optimization and/or second-order correction.
 Total Energy, E(TD-HF/TD-DFT) =  -741.559397638
 Copying the excited state density for this state as the 1-particle RhoCI density.

 Excited State   2:  3.000-A"     2.2457 eV  552.10 nm  f=0.0000  <S**2>=2.000
     56A -> 61A        0.14851
     59A -> 61A        0.67409
     56B -> 61B       -0.14851
     59B -> 61B       -0.67409

 Excited State   3:  1.000-A'     2.2695 eV  546.31 nm  f=0.0269  <S**2>=0.000
     60A -> 61A        0.70393
     60B -> 61B        0.70393

 Excited State   4:  3.000-A'     2.4995 eV  496.04 nm  f=0.0000  <S**2>=2.000
     57A -> 61A        0.28047
     58A -> 61A        0.56077
     60A -> 62A       -0.18313
     60A -> 63A        0.22825
     57B -> 61B       -0.28047
     58B -> 61B       -0.56077
     60B -> 62B        0.18313
     60B -> 63B       -0.22825
```

[…]

S3.2. TD-B3LYP-D3: Dexcitation energies and orbital contribution in the fluorescence spectra computed for S_1

Tetracene

60 HOMO, 61 LUMO

```
Excited State   1:  3.000-A'     0.6679 eV 1856.45 nm  f=0.0000  <S**2>=2.000
     59A -> 62A       -0.12750
     60A -> 61A        0.75647
     59B -> 62B        0.12750
     60B -> 61B       -0.75647
     60A <- 61A        0.30319
     60B <- 61B       -0.30319

 Excited State   2:  1.000-A'     2.0810 eV  595.79 nm  f=0.0419  <S**2>=0.000
     60A -> 61A        0.70993
     60B -> 61B        0.70993
 This state for optimization and/or second-order correction.
 Total Energy, E(TD-HF/TD-DFT) =  -693.399178093
 Copying the excited state density for this state as the 1-particle RhoCI density.

 Excited State   3:  3.000-A'     2.2831 eV  543.06 nm  f=0.0000  <S**2>=2.000
     59A -> 61A       -0.53374
     60A -> 62A        0.44404
     59B -> 61B        0.53374
     60B -> 62B       -0.44404
```

```
 Excited State   4:  3.000-A'     3.0965 eV  400.40 nm  f=0.0000  <S**2>=2.000
     58A -> 61A        0.65291
     60A -> 63A        0.25565
     58B -> 61B       -0.65291
     60B -> 63B       -0.25565
```

[…]

NH2T

68 HOMO, 69 LUMO

```
Excited State   1:  3.000-A      0.1006 eV 12329.36 nm  f=0.0000  <S**2>=2.000
     66A -> 71A       -0.10769
     67A -> 69A        0.13551
     67A -> 70A       -0.13302
     68A -> 69A        1.18078
     68A -> 70A        0.13799
     68A -> 79A       -0.14498
     66B -> 71B        0.10769
     67B -> 69B       -0.13551
     67B -> 70B        0.13302
     68B -> 69B       -1.18078
     68B -> 70B       -0.13799
     68B -> 79B        0.14498
     66A <- 71A       -0.10692
     67A <- 69A        0.12754
     67A <- 70A       -0.12342
     68A <- 69A        0.95424
     68A <- 70A        0.12321
     68A <- 79A       -0.13792
     66B <- 71B        0.10692
     67B <- 69B       -0.12754
     67B <- 70B        0.12342
     68B <- 69B       -0.95424
     68B <- 70B       -0.12321
     68B <- 79B        0.13792

 Excited State   2:  1.000-A      1.3213 eV  938.37 nm  f=0.0313  <S**2>=0.000
     68A -> 69A        0.71276
     68B -> 69B        0.71276
     68A <- 69A       -0.11304
     68B <- 69B       -0.11304
 This state for optimization and/or second-order correction.
 Total Energy, E(TD-HF/TD-DFT) =  -804.185599338
 Copying the excited state density for this state as the 1-particle RhoCI density.

 Excited State   3:  3.000-A      2.0004 eV  619.81 nm  f=0.0000  <S**2>=2.000
     67A -> 69A       -0.41365
     68A -> 70A        0.55056
     67B -> 69B        0.41365
     68B -> 70B       -0.55056

 Excited State   4:  3.000-A      2.3696 eV  523.23 nm  f=0.0000  <S**2>=2.000
     68A -> 71A        0.70121
     68B -> 71B       -0.70121
```

[…]

NMe2T

84 HOMO, 85 LUMO

```
Excited State   1:  3.000-A      0.4641 eV 2671.32 nm  f=0.0000  <S**2>=2.000
     83A -> 85A        0.11468
     84A -> 85A        0.77153
     83B -> 85B       -0.11468
     84B -> 85B       -0.77153
     84A <- 85A        0.34353
     84B <- 85B       -0.34353

 Excited State   2:  1.000-A      1.6263 eV  762.39 nm  f=0.0707  <S**2>=0.000
     84A -> 85A        0.70920
     84B -> 85B        0.70920
 This state for optimization and/or second-order correction.
 Total Energy, E(TD-HF/TD-DFT) =  -961.457042293
 Copying the excited state density for this state as the 1-particle RhoCI density.

 Excited State   3:  3.000-A      2.0667 eV  599.92 nm  f=0.0000  <S**2>=2.000
     81A -> 85A       -0.18771
     83A -> 85A       -0.54727
     83A -> 86A        0.12588
     84A -> 86A        0.36756
     81B -> 85B        0.18771
     83B -> 85B        0.54727
     83B -> 86B       -0.12588
     84B -> 86B       -0.36756

 Excited State   4:  3.000-A      2.5857 eV  479.50 nm  f=0.0000  <S**2>=2.000
     81A -> 85A       -0.24469
     81A -> 86A       -0.12745
     83A -> 85A        0.40531
     84A -> 86A        0.48484
     81B -> 85B        0.24469
     81B -> 86B        0.12745
     83B -> 85B       -0.40531
     84B -> 86B       -0.48484
```

[…]

NPh2OMeT

180 HOMO, 181 LUMO

```
Excited State   1:  3.000-A      0.7541 eV 1644.03 nm  f=0.0000  <S**2>=2.000
    178A -> 181A       0.43167
    179A -> 181A       0.36617
    180A -> 181A      -0.46736
    178B -> 181B      -0.43167
    179B -> 181B      -0.36617
    180B -> 181B       0.46736
    178A <- 181A       0.17277
    179A <- 181A       0.11751
    180A <- 181A      -0.11568
    178B <- 181B      -0.17277
    179B <- 181B      -0.11751
    180B <- 181B       0.11568

 Excited State   2:  1.000-A      1.4005 eV  885.31 nm  f=0.0478  <S**2>=0.000
    179A -> 181A       0.11469
    180A -> 181A       0.69365
    179B -> 181B       0.11469
    180B -> 181B       0.69365
 This state for optimization and/or second-order correction.
```

```
 Total Energy, E(TD-HF/TD-DFT) =  -2187.11332645
 Copying the excited state density for this state as the 1-particle RhoCI density.

 Excited State   3:  3.000-A      1.4165 eV  875.27 nm  f=0.0000  <S**2>=2.000
    178A -> 181A      -0.22766
    179A -> 181A      -0.41312
    180A -> 181A      -0.52286
    178B -> 181B       0.22766
    179B -> 181B       0.41312
    180B -> 181B       0.52286

 Excited State   4:  3.000-A      1.7009 eV  728.93 nm  f=0.0000  <S**2>=2.000
    178A -> 181A       0.52105
    179A -> 181A      -0.44799
    180A -> 181A       0.12072
    178B -> 181B      -0.52105
    179B -> 181B       0.44799
    180B -> 181B      -0.12072
```

[…]

NO2T

82 HOMO, 83 LUMO

```
Excited State   1:  3.000-A      0.5692 eV 2178.30 nm  f=0.0000  <S**2>=2.000
     82A -> 83A       -0.73035
     82A -> 84A        0.17217
     82B -> 83B        0.73035
     82B -> 84B       -0.17217
     82A <- 83A       -0.27032
     82A <- 84A        0.10341
     82B <- 83B        0.27032
     82B <- 84B       -0.10341

 Excited State   2:  1.000-A      1.6077 eV  771.21 nm  f=0.0460  <S**2>=0.000
     82A -> 83A        0.70423
     82B -> 83B        0.70423
 This state for optimization and/or second-order correction.
 Total Energy, E(TD-HF/TD-DFT) =  -1102.58122410
 Copying the excited state density for this state as the 1-particle RhoCI density.

 Excited State   3:  3.000-A      1.9676 eV  630.13 nm  f=0.0000  <S**2>=2.000
     81A -> 83A       -0.46764
     81A -> 84A        0.10145
     82A -> 84A        0.45404
     82A -> 86A       -0.21693
     81B -> 83B        0.46764
     81B -> 84B       -0.10145
     82B -> 84B       -0.45404
     82B -> 86B        0.21693

 Excited State   4:  3.000-A      2.2983 eV  539.46 nm  f=0.0000  <S**2>=2.000
     81A -> 83A       -0.45324
     81A -> 86A        0.10772
     82A -> 83A       -0.16621
     82A -> 84A       -0.48691
     81B -> 83B        0.45324
     81B -> 86B       -0.10772
     82B -> 83B        0.16621
     82B -> 84B        0.48691
```

[…]

DAT

60 HOMO, 61 LUMO

```
Excited State   1:  3.000-A'     0.6809 eV 1820.78 nm  f=0.0000  <S**2>=2.000
     59A -> 62A        0.11251
     60A -> 61A       -0.74587
     59B -> 62B       -0.11251
     60B -> 61B        0.74587
     60A <- 61A       -0.28502
     60B <- 61B        0.28502

 Excited State   2:  1.000-A'     1.9704 eV  629.25 nm  f=0.0276  <S**2>=0.000
     60A -> 61A        0.70717
     60B -> 61B        0.70717
 This state for optimization and/or second-order correction.
 Total Energy, E(TD-HF/TD-DFT) =  -725.486202395
 Copying the excited state density for this state as the 1-particle RhoCI density.

 Excited State   3:  3.000-A'     2.2278 eV  556.54 nm  f=0.0000  <S**2>=2.000
     59A -> 61A       -0.60608
     60A -> 62A        0.33964
     59B -> 61B        0.60608
     60B -> 62B       -0.33964

 Excited State   4:  3.000-A"     2.2614 eV  548.26 nm  f=0.0000  <S**2>=2.000
     58A -> 61A       -0.69049
     58B -> 61B        0.69049
```

[…]

TrAT1

60 HOMO, 61 LUMO

```
Excited State   1:  3.000-A'     0.6965 eV 1780.06 nm  f=0.0000  <S**2>=2.000
     57A -> 61A       -0.10462
     60A -> 61A       -0.73522
     60A -> 62A       -0.12482
     57B -> 61B        0.10462
     60B -> 61B        0.73522
     60B -> 62B        0.12482
     60A <- 61A       -0.26715
     60B <- 61B        0.26715

 Excited State   2:  1.000-A'     1.9019 eV  651.91 nm  f=0.0220  <S**2>=0.000
     60A -> 61A        0.70636
     60B -> 61B        0.70636

 Excited State   3:  3.000-A"     2.0082 eV  617.38 nm  f=0.0000  <S**2>=2.000
     59A -> 61A        0.68802
     59B -> 61B       -0.68802
 This state for optimization and/or second-order correction.
 Total Energy, E(TD-HF/TD-DFT) =  -741.523990456
 Copying the excited state density for this state as the 1-particle RhoCI density.

 Excited State   4:  1.000-A"     2.3404 eV  529.76 nm  f=0.0001  <S**2>=0.000
     59A -> 61A        0.70251
     59B -> 61B        0.70251
```

[…]

TrAT2

60 HOMO, 61 LUMO

```
Excited State   1:  3.000-A'     0.6414 eV 1933.17 nm  f=0.0000  <S**2>=2.000
     60A -> 61A       -0.74841
     60B -> 61B        0.74841
     60A <- 61A       -0.29367
     60B <- 61B        0.29367

 Excited State   2:  1.000-A'     1.8960 eV  653.93 nm  f=0.0251  <S**2>=0.000
     60A -> 61A        0.70679
     60B -> 61B        0.70679

 Excited State   3:  3.000-A"     2.1682 eV  571.83 nm  f=0.0000  <S**2>=2.000
     56A -> 61A        0.19262
     59A -> 61A        0.66345
     56B -> 61B       -0.19262
     59B -> 61B       -0.66345
 This state for optimization and/or second-order correction.
 Total Energy, E(TD-HF/TD-DFT) =  -741.515839951
 Copying the excited state density for this state as the 1-particle RhoCI density.

 Excited State   4:  3.000-A'     2.2090 eV  561.28 nm  f=0.0000  <S**2>=2.000
     57A -> 61A       -0.15401
     58A -> 61A       -0.59716
     60A -> 62A        0.30855
     57B -> 61B        0.15401
     58B -> 61B        0.59716
     60B -> 62B       -0.30855
```

[…]

S4. Supplementary theoretical UV-Vis absorption data for the monomers: natural transition orbitals analysis for the $S_0 \rightarrow T_1$, $S_0 \rightarrow S_1$, and $S_0 \rightarrow T_2$ transitions

NTOs were computed to characterize the orbital nature of the low-lying singlet and triplet excited states and to assess the dominant electronic transitions relevant to TTA-UC in addition to the difference density maps reported in Figure S2-Figure S4.

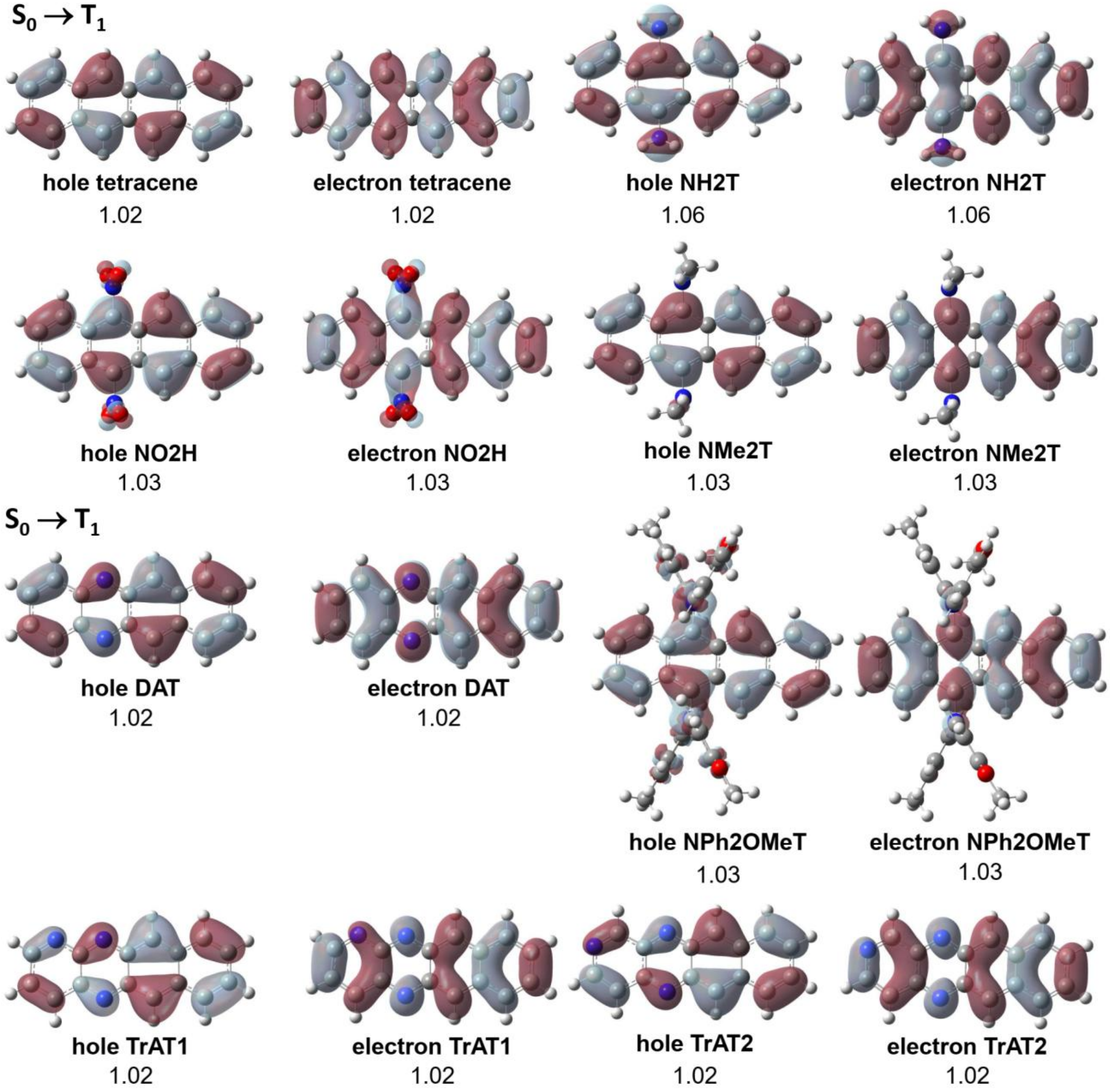

Figure S6. Dominant natural transition orbitals (NTOs) for the $S_0 \rightarrow T_1$ excitation of the investigated tetracene derivatives, computed at the TD-B3LYP-D3/def2-TZVP level. For

each molecule, the dominant hole and particle NTOs are shown together with their corresponding weights. The reported NTO weights quantify the relative importance of the leading orbital contributions. Isosurface: 0.02 a.u. Grey regions: positive values, purple regions: negative values. Color code: grey (carbon), white (hydrogen), blue (nitrogen).

In all cases, the lowest triplet state is mainly characterized by a π–π* single-excitation character, while differences in orbital localization and substituent participation are observed among the derivatives. The $S_0 \rightarrow S_1$ transition is predominantly described by a single π–π* excitation localized on the tetracene core, with limited substituent involvement, besides for NPh2OMeT.

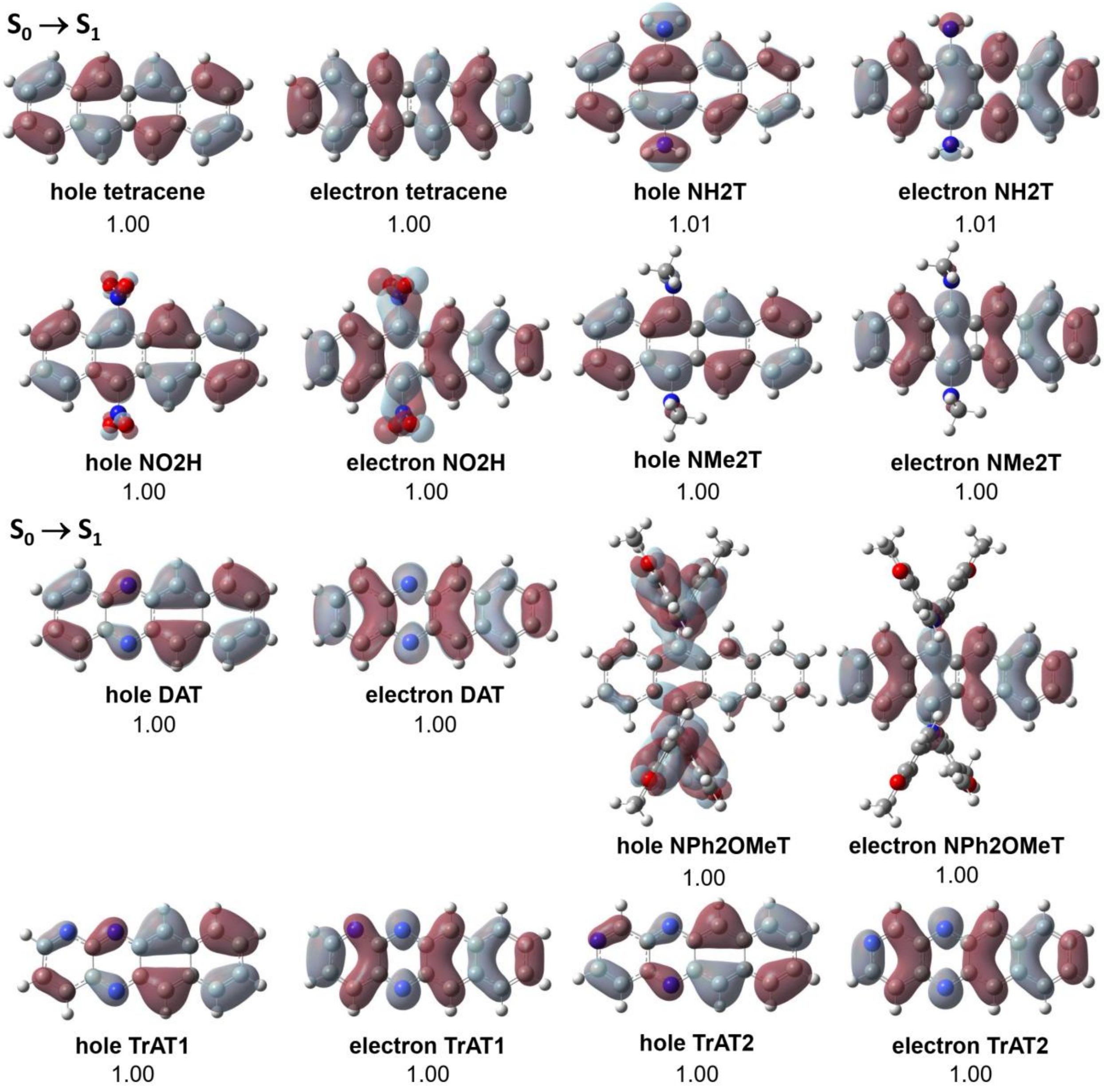

Figure S7. Dominant natural transition orbitals (NTOs) for the $S_0 \rightarrow S_1$ excitation of the investigated tetracene derivatives, computed at the TD-B3LYP-D3/def2-TZVP level. For each molecule, the dominant hole and particle NTOs are shown together with their corresponding weights. Isosurface: 0.02 a.u. Grey regions: positive values, purple regions: negative values. Color code: grey (carbon), white (hydrogen), blue (nitrogen).

Compared to T_1, the T_2 state exhibits a more heterogeneous orbital character (see Figure S4), with two hole–particle NTO pairs contributing to the transition density in several compounds, reflecting increased orbital mixing at higher excitation energies.

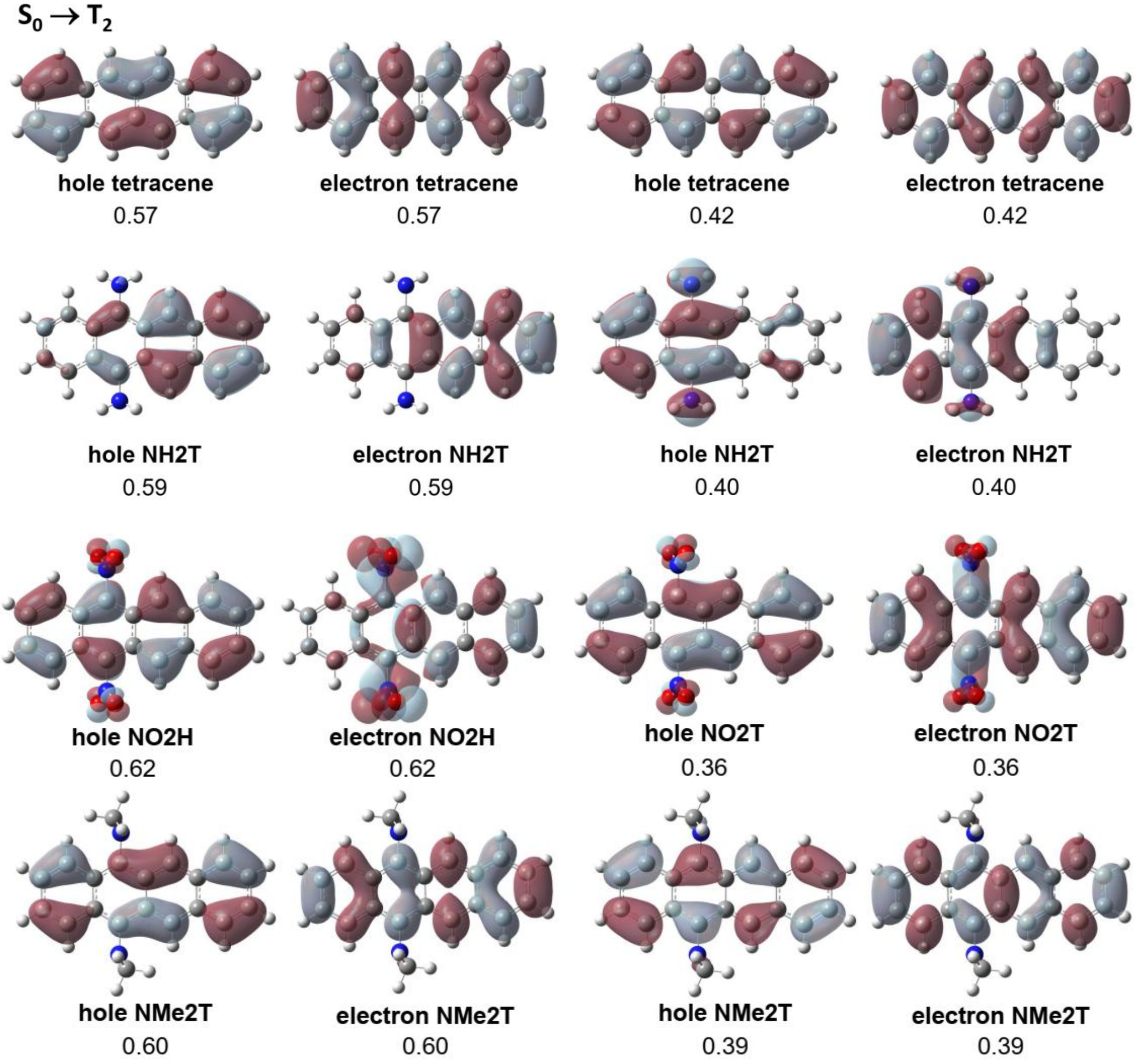

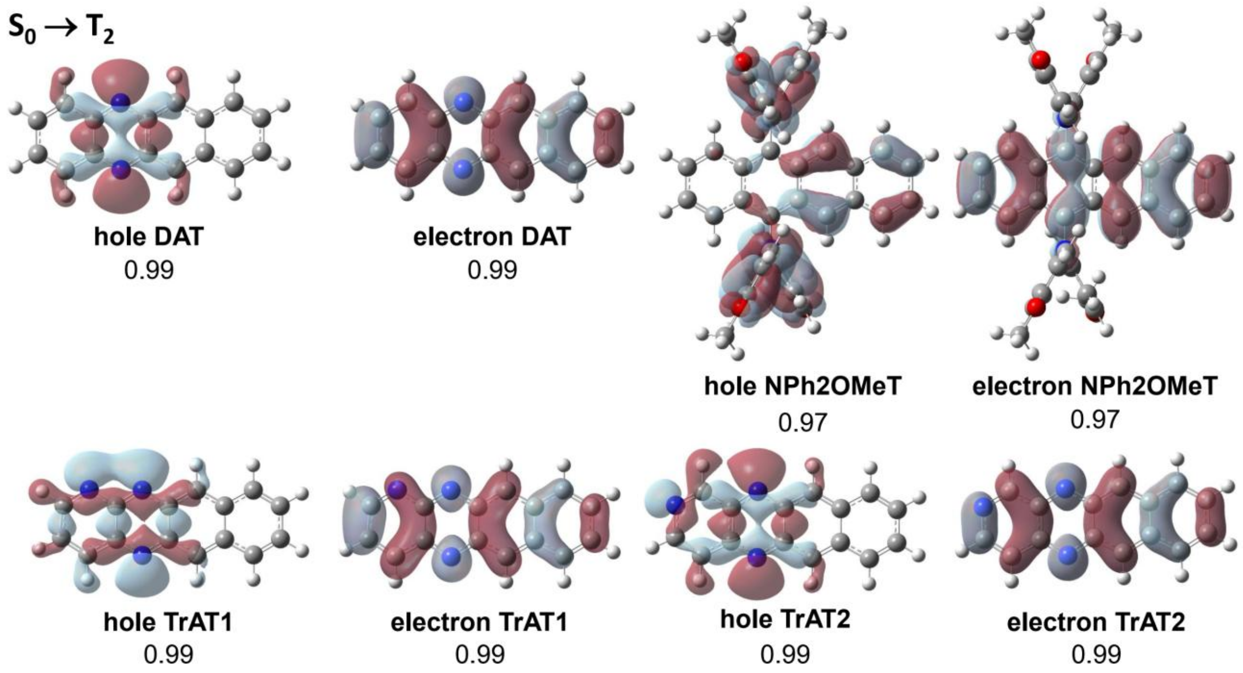

Figure S8. Dominant natural transition orbitals (NTOs) for the $S_0 \rightarrow T_2$ excitation of the investigated tetracene derivatives, computed at the TD-B3LYP-D3/def2-TZVP level. For each molecule, the dominant hole and particle NTOs are shown together with their corresponding weights. Isosurface: 0.02 a.u. Grey regions: positive values, purple regions: negative values. Color code: grey (carbon), white (hydrogen), blue (nitrogen).

S5. Supplementary theoretical UV-Vis absorption data in dichloromethane

Table S3. Computed electronic properties of the investigated emitters at the TD-B3LYP-D3 level of theory in dichloromethane as implicit solvent. For all the transitions, the vertical excitation energies (ΔE_{vert}), together with the corresponding absorption wavelengths (λ_{abs}, nm), oscillator strengths (f, a.u.), transition dipole moments (μ, a.u.) are reported. All energies are reported in eV unless otherwise specified.

	T	NH2T	NMe2T	NPh2OMeT	DAT	TrAT1	TrAT2
$S_0 \rightarrow S_1$							
$\Delta E_{vertS0\rightarrow S1}$	2.40	1.81	2.32	1.71	2.27	2.20	2.16
$\lambda_{absS0\rightarrow S1}$	517	687	534	726	547	565	574
$f_{S0\rightarrow S1}$	0.06	0.07	0.09	0.14	0.04	0.03	0.03
$\mu_{S0\rightarrow S1}$	0.10	0.10	0.14	0.21	0.06	0.05	0.05
Excitation state n.	2	2	2	2	2	2	2
$S_0 \rightarrow T_1$							
$\Delta E_{vertS0\rightarrow T1}$	1.17	0.83	1.11	1.00	1.18	1.20	1.13
$\lambda_{absS0\rightarrow T1}$	1057	1492	1115	1234	1051	1037	1095
Excitation state n.	1	1	1	1	1	1	1
$S_0 \rightarrow T_2$							
$\Delta E_{vertS0\rightarrow T2}$	2.55	2.35	2.50	1.86	2.45	2.28	2.32
$\lambda_{absS0\rightarrow T2}$	486	528	495	666	507	544	534
Excitation state n.	3	3	3	3	3	3	3

S6. Supplementary theoretical UV-Vis absorption data at the TD-CAM-B3LYP-D3 level of theory

Table S4. Computed electronic properties of the investigated emitters at the TD-CAM-B3LYP-D3 level of theory. For all the transitions, the vertical excitation energies (ΔE_{vert}), together with the corresponding absorption wavelengths (λ_{abs}, nm), oscillator strengths (f, a.u.), transition dipole moments (μ, a.u.) are reported. All energies are reported in eV unless otherwise specified.

	T	NH2T	NMe2T	NPh2OMeT	NO2T	DAT	TrAT1	TrAT2
$S_0 \rightarrow S_1$								
$\Delta E_{vertS0 \rightarrow S1}$	2.76	2.18	2.67	2.32	2.62	2.71	2.70	2.66
$\lambda_{absS0 \rightarrow S1}$	450	569	464	535	473	457	460	466
$f_{S0 \rightarrow S1}$	0.07	0.08	0.11	0.20	0.09	0.05	0.04	0.05
$\mu_{S0 \rightarrow S1}$	0.11	0.12	0.17	0.31	0.14	0.08	0.07	0.08
Excitation state n.	3	2	3	3	3	4	4	4
$S_0 \rightarrow T_1$								
$\Delta E_{vertS0 \rightarrow T1}$	0.81	0.37	0.73	0.65	0.71	0.90	0.95	0.88
$\lambda_{absS0 \rightarrow T1}$	1527	3325	1697	1898	1741	1384	1311	1403
Excitation state n.	1	1	1	1	1	1	1	1
$S_0 \rightarrow T_2$								
$\Delta E_{vertS0 \rightarrow T2}$	2.45	2.29	2.41	2.26	2.37	2.45	2.49	2.48
$\lambda_{absS0 \rightarrow T2}$	506	542	515	549	523	506	498	501
Excitation state n.	2	3	2	2	2	2	2	2

S7. Supplementary data on the dimers

The optimized structures of the tetracene and NH2T dimers in the fundamental state are reported in Figure S9.

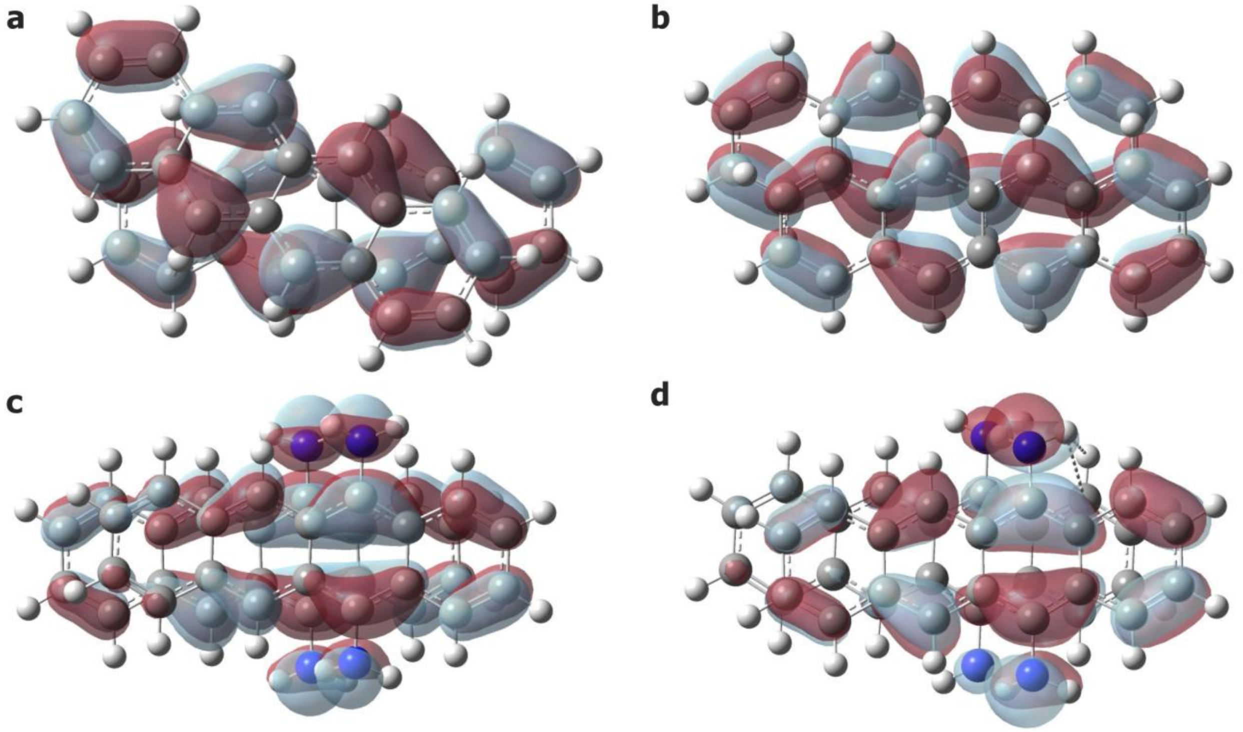

Figure S9. Orbital overlap in the dimers in S_0. Optimized geometries for **a**. **tetracene-2S**, **b**. **tetracene-2C**, **c**. **NH2T-2S**, and **d**. **NH2T-2C** obtained at the B3LYP-D3BJ/def2-TZVP level of theory in the ground state and in the gas phase. The HOMO orbitals are reported. Grey regions: positive values, purple regions: negative values. Color code: grey (carbon), white (hydrogen), blue (nitrogen). The corresponding side view of the dimers is reported in Figure 5.

The HOMO and LUMO orbitals for the Q_1 state of tetracene-2S are reported in Figure S10.

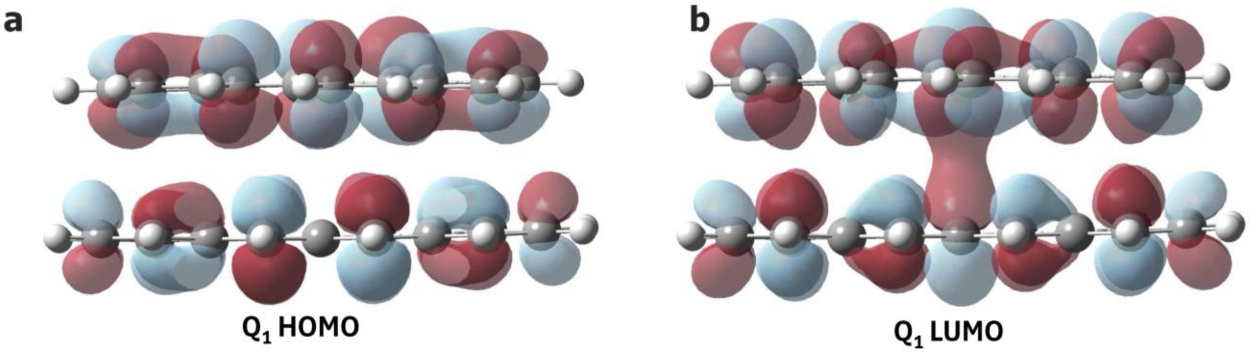

Figure S10. Frontier orbitals in the tetracene-2S dimer in Q_1. Data obtained at the B3LYP-D3BJ/def2-TZVP level of theory in the gas phase. The HOMO (**a**) and LUMO (**b**) orbitals are reported. Grey regions: positive values, purple regions: negative values. Color code: grey (carbon), white (hydrogen).

Table S5. Geometrical and electronic properties of tetracene and NH2T dimers in ground and excited states. Maximal interplanar distance between the monomers (*d*) and the average absolute distance (d_{avg}) of the atoms from the mean plane defined by the tetracene core for each monomer are reported in Å. Atomic charges (*q*) and spin densities (*ρ*) on the tetracene core (C atoms only) are given for all emitters (in a.u.). 1 and 2 in the subscript refer to the different monomers values. Values obtained at the (TD-)B3LYP-D3/def2-TZVP level of theory.

	tetracene-2S	tetracene-2C	NH2T-2S	NH2T-2C
**S_0**				
d	3.378	3.369	3.670	3.854
d_{avg1}	0.021	0.006	0.024	0.020
d_{avg2}	0.021	0.006	0.032	0.078
$\rho_{Hirshfeld1}$	0.00	0.00	0.00	0.00
$q_{Hirshfeld1}$	-0.46	-0.46	-0.41	-0.39
q_{CM51}	-1.14	-1.14	-0.86	-0.84
$\rho_{Hirshfeld2}$	0.00	0.00	0.00	0.00
$q_{Hirshfeld2}$	-0.46	-0.46	-0.41	-0.43
q_{CM52}	-1.14	-1.14	-0.86	-0.88
**S_1**				
d	3.395	3.264	3.634	4.360
d_{avg1}	0.043	0.011	0.053	0.046
d_{avg2}	0.043	0.011	0.053	0.200
$\rho_{Hirshfeld1}$	0.00	0.00	0.00	0.00
$q_{Hirshfeld1}$	-0.45	-0.45	-0.42	-0.35
q_{CM51}	-1.14	-1.14	-0.86	-0.80
$\rho_{Hirshfeld2}$	0.00	0.00	0.00	0.00
$q_{Hirshfeld2}$	-0.45	-0.45	-0.42	-0.50
q_{CM52}	-1.14	-1.14	-0.86	-0.93
**$T_{1\ A(NO\ TD)}$**				
d	3.272	3.272	3.548	3.755
d_{avg1}	0.005	0.005	0.047	0.019
d_{avg2}	0.005	0.005	0.029	0.092
$\rho_{Hirshfeld1}$	0.95	0.95	0.79	0.94
$q_{Hirshfeld1}$	-0.45	-0.45	-0.43	-0.55

q_{CM51}	-1.14	-1.14	-0.87	-1.00
$\rho_{Hirshfeld2}$	0.95	0.95	0.83	0.69
$q_{Hirshfeld2}$	-0.45	-0.45	-0.45	-0.33
q_{CM52}	-1.14	-1.14	-0.89	-0.77
**T_1 B(NO TD)**				
d	3.448	3.370	3.693	3.899
d_{avg1}	0.014	0.018	0.037	0.012
d_{avg2}	0.028	0.010	0.023	0.092
$\rho_{Hirshfeld1}$	0.05	0.11	0.04	0.10
$q_{Hirshfeld1}$	-0.46	-0.49	-0.41	-0.43
q_{CM51}	-1.14	-1.17	-0.85	-0.87
$\rho_{Hirshfeld2}$	1.85	1.79	1.61	1.52
$q_{Hirshfeld2}$	-0.45	-0.42	-0.47	-0.46
q_{CM52}	-1.13	-1.11	-0.91	-0.89
**Q_1**				
d	3.387	3.373	3.776	3.847
d_{avg1}	0.029	0.010	0.034	0.047
d_{avg2}	0.029	0.009	0.020	0.094
$\rho_{Hirshfeld1}$	1.90	1.90	1.65	1.67
$q_{Hirshfeld1}$	-0.45	-0.45	-0.47	-0.45
q_{CM51}	-1.14	-1.14	-0.90	-0.89
$\rho_{Hirshfeld2}$	1.90	1.90	1.65	1.64
$q_{Hirshfeld2}$	-0.45	-0.45	-0.46	-0.48
q_{CM52}	-1.14	-1.13	-0.90	-0.91

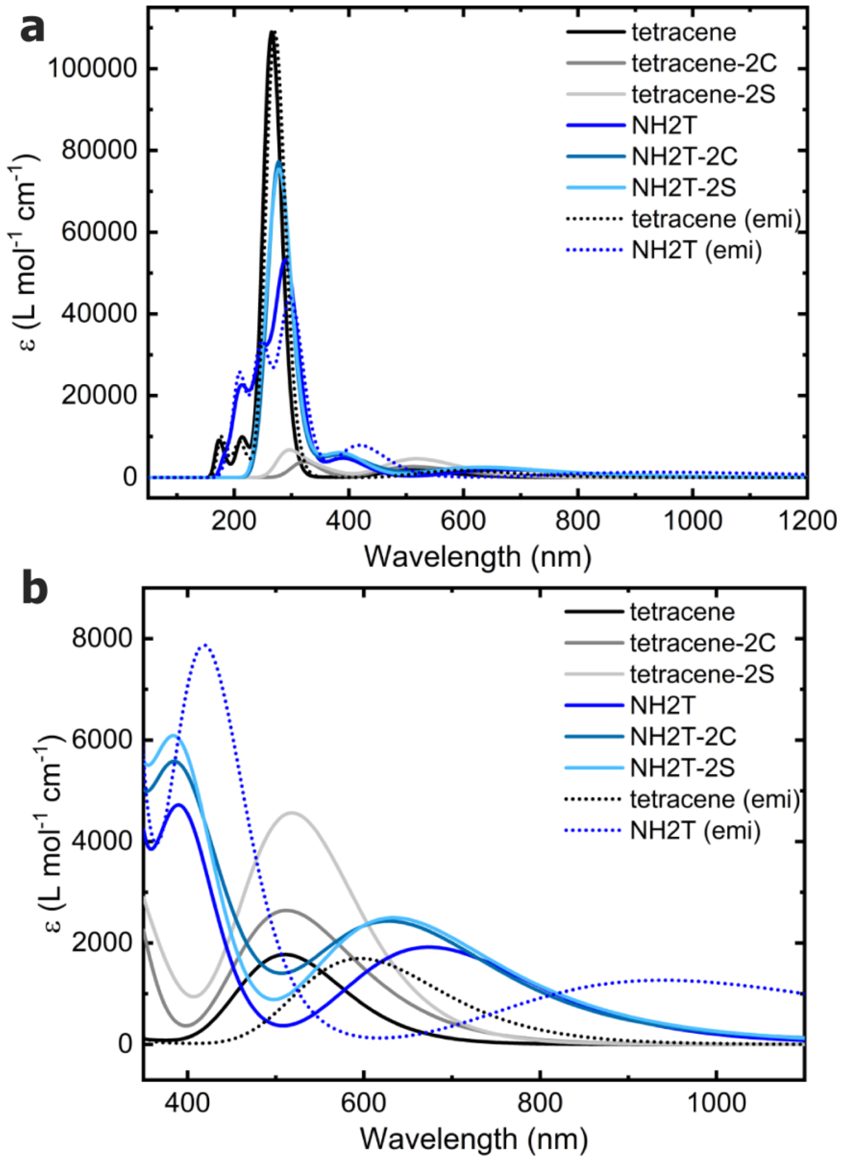

Figure S11. Spectroscopic features of the most red-shifted TTA-UC chromophore. TD-B3LYP-D3/def2-TZVP computed UV–Vis absorption spectra of NH2T in its monomeric form (blue solid line) and in two dimeric configurations, NH2T-2C and NH2T-2S (light-blue lines). For comparison, the spectra of tetracene are also reported, including the monomer (black solid line) and the dimeric species tetracene-2C (dark grey) and tetracene-2S (light grey). Emission spectra are shown as dashed lines for both NH2T (blue) and tetracene (black). **a.** Full spectral range. **b.** A magnified view of the 350–1100 nm spectral region.

The difference of the total electronic density of T_1 and S_1 and the ground state of tetracene and NH2T dimers as computed at the TD-B3LYP-D3/def2-TZVP are reported in Figure S12 and Figure S13, respectively.

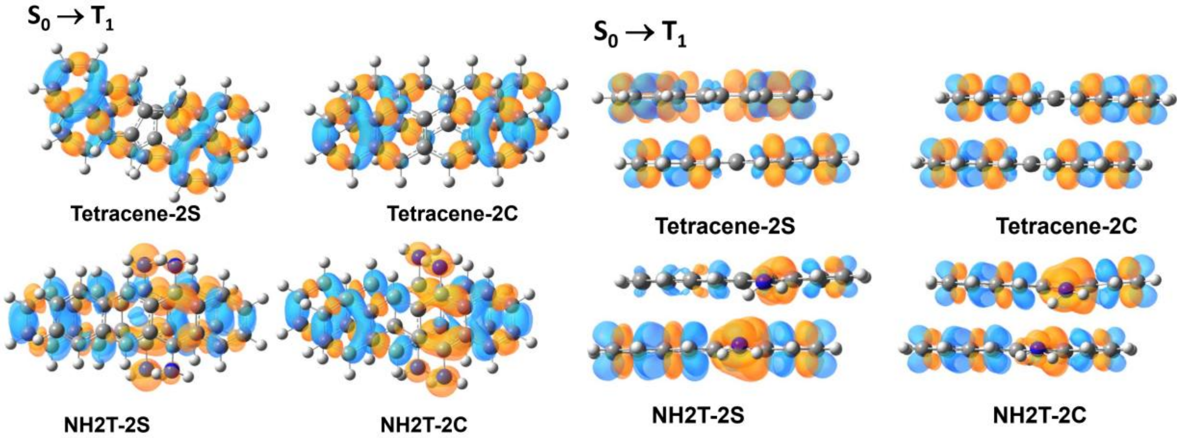

Figure S12. Difference of the total electronic density of the first excited triplet state and the ground state of tetracene and NH2T dimers. as computed at the TD-B3LYP-D3/def2-TZVP including the first 100 (de)excitations. **Left.** Top view. **Right.** Side view. Blue regions: positive values, corresponding to an increase of the total electronic density in the excited state with respect to S_0. Orange regions: negative values. Isosurface at 0.0006 a.u. Color code of the atoms: blue (nitrogen), grey (carbon), white (hydrogen).

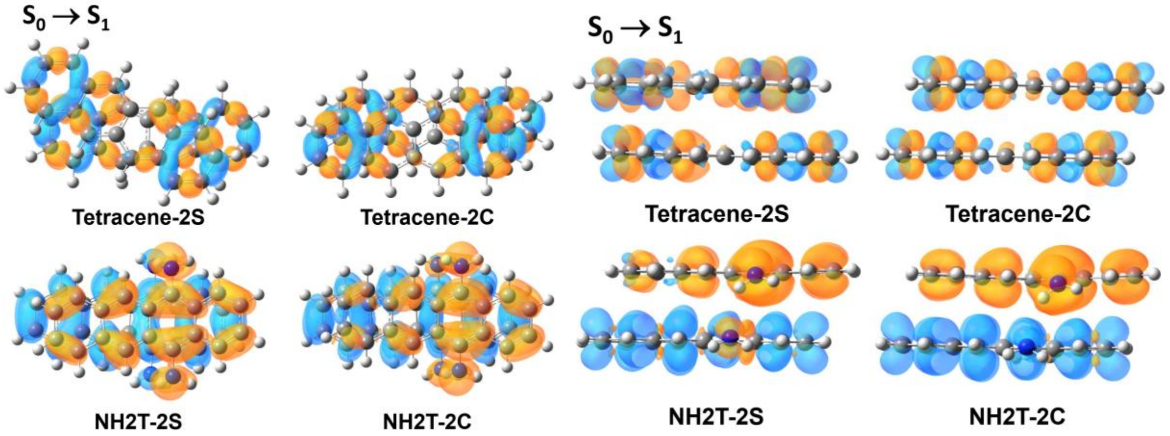

Figure S13. Difference of the total electronic density of the first excited singlet state and the ground state of tetracene and NH2T dimers. as computed at the TD-B3LYP-D3/def2-TZVP including the first 100 (de)excitations. **Left.** Top view. **Right.** Side view. Blue regions: positive values, corresponding to an increase of the total electronic density in the excited state with respect to S_0. Orange regions: negative values. Isosurface at 0.0006 a.u. Color code of the atoms: blue (nitrogen), grey (carbon), white (hydrogen).

S7.1. TD-B3LYP-D3: Excitation energies and orbital contribution in the absorption spectra computed for S_0

NH2T dimers 2S

136 HOMO, 137 LUMO

```
Excited State   1:  3.000-A      0.8459 eV 1465.67 nm  f=0.0000  <S**2>=2.000
    135A -> 137A       0.13595
    135A -> 138A       0.37441
    136A -> 137A      -0.57762
    136A -> 138A      -0.15075
    135B -> 137B      -0.13595
    135B -> 138B      -0.37441
    136B -> 137B       0.57762
    136B -> 138B       0.15075
    135A <- 138A       0.11301
    136A <- 137A      -0.14097
    135B <- 138B      -0.11301
    136B <- 137B       0.14097
 This state for optimization and/or second-order correction.
 Total Energy, E(TD-HF/TD-DFT) =  -1608.49531470
 Copying the excited state density for this state as the 1-particle RhoCI density.
```

```
 Excited State   2:  3.000-A      0.8966 eV 1382.81 nm  f=0.0000  <S**2>=2.000
    135A -> 137A      -0.43876
    135A -> 138A       0.17673
    136A -> 137A      -0.12545
    136A -> 138A       0.52120
    135B -> 137B       0.43876
    135B -> 138B      -0.17673
    136B -> 137B       0.12545
    136B -> 138B      -0.52120
    135A <- 137A      -0.11782
    136A <- 138A       0.12246
    135B <- 137B       0.11782
    136B <- 138B      -0.12246

 Excited State   3:  1.000-A      1.6300 eV  760.65 nm  f=0.0097  <S**2>=0.000
    135A -> 138A       0.21309
    136A -> 137A       0.57595
    136A -> 138A      -0.33590
    135B -> 138B       0.21309
    136B -> 137B       0.57595
    136B -> 138B      -0.33590

 Excited State   4:  3.000-A      1.6726 eV  741.27 nm  f=0.0000  <S**2>=2.000
    135A -> 137A      -0.31120
    135A -> 138A       0.43665
    136A -> 137A       0.29921
    136A -> 138A      -0.33940
    135B -> 137B       0.31120
    135B -> 138B      -0.43665
    136B -> 137B      -0.29921
    136B -> 138B       0.33940
```

[…]

NH2T dimers 2C

136 HOMO, 137 LUMO

```
Excited State   1:  3.000-A      0.8227 eV 1506.98 nm  f=0.0000  <S**2>=2.000
    135A -> 137A       0.36494
    135A -> 138A       0.13099
    136A -> 137A       0.35608
    136A -> 138A      -0.48969
    135B -> 137B      -0.36494
    135B -> 138B      -0.13099
    136B -> 137B      -0.35608
    136B -> 138B       0.48969
    135A <- 137A       0.11051
    136A <- 138A      -0.13398
    135B <- 137B      -0.11051
    136B <- 138B       0.13398
 This state for optimization and/or second-order correction.
 Total Energy, E(TD-HF/TD-DFT) =  -1608.49394227
 Copying the excited state density for this state as the 1-particle RhoCI density.

 Excited State   2:  3.000-A      0.8804 eV 1408.30 nm  f=0.0000  <S**2>=2.000
    135A -> 137A      -0.46884
    136A -> 137A      -0.21684
    136A -> 138A      -0.49126
    135B -> 137B       0.46884
    136B -> 137B       0.21684
    136B -> 138B       0.49126
    135A <- 137A      -0.13016
```

```
   136A <- 138A       -0.11978
   135B <- 137B        0.13016
   136B <- 138B        0.11978

 Excited State   3:  1.000-A      1.3107 eV  945.95 nm  f=0.0022  <S**2>=0.000
   136A -> 137A        0.70138
   136B -> 137B        0.70138

 Excited State   4:  3.000-A      1.3709 eV  904.39 nm  f=0.0000  <S**2>=2.000
   135A -> 137A        0.39105
   136A -> 137A       -0.57225
   136A -> 138A       -0.11356
   135B -> 137B       -0.39105
   136B -> 137B        0.57225
   136B -> 138B        0.11356

 Excited State   5:  1.000-A      1.7766 eV  697.86 nm  f=0.0137  <S**2>=0.000
   135A -> 137A        0.14225
   135A -> 138A        0.10661
   136A -> 138A        0.68314
   135B -> 137B        0.14225
   135B -> 138B        0.10661
   136B -> 138B        0.68314
```

[…]

Tetracene dimer 2S

120 HOMO, 121 LUMO

```
Excited State   1:      Triplet-A      1.2260 eV 1011.31 nm  f=0.0000  <S**2>=2.000
     119 -> 121        0.51175
     120 -> 122       -0.48910
     119 <- 121        0.11914
     120 <- 122       -0.11941
 This state for optimization and/or second-order correction.
 Total Energy, E(TD-HF/TD-DFT) =  -1386.94721491
 Copying the excited state density for this state as the 1-particle RhoCI density.

 Excited State   2:      Triplet-A      1.2721 eV  974.68 nm  f=0.0000  <S**2>=2.000
     119 -> 122       -0.46146
     120 -> 121        0.53439
     119 <- 122       -0.10426
     120 <- 121        0.11078

 Excited State   3:      Triplet-A      2.1490 eV  576.95 nm  f=0.0000  <S**2>=2.000
     119 -> 122        0.51529
     120 -> 121        0.44434

 Excited State   4:      Triplet-A      2.1698 eV  571.41 nm  f=0.0000  <S**2>=2.000
     119 -> 121        0.48774
     120 -> 122        0.50982

 Excited State   5:      Singlet-A      2.1924 eV  565.52 nm  f=0.0016  <S**2>=0.000
     119 -> 122        0.39610
     120 -> 121        0.58492
```

[…]

Tetracene dimer 2C

120 HOMO, 121 LUMO

```
Excited State   1:      Triplet-AU     1.1573 eV 1071.30 nm  f=0.0000  <S**2>=2.000
     119 -> 122        0.43768
     120 -> 121        0.56015
     119 <- 122        0.11738
     120 <- 121        0.12947
 This state for optimization and/or second-order correction.
 Total Energy, E(TD-HF/TD-DFT) =  -1386.94775761
 Copying the excited state density for this state as the 1-particle RhoCI density.

 Excited State   2:      Triplet-AG     1.1932 eV 1039.12 nm  f=0.0000  <S**2>=2.000
     119 -> 121        0.50660
     120 -> 122        0.49549
     119 <- 121        0.11902
     120 <- 122        0.11907

 Excited State   3:      Triplet-AU     2.1280 eV  582.64 nm  f=0.0000  <S**2>=2.000
     119 -> 122        0.53748
     120 -> 121       -0.42002

 Excited State   4:      Singlet-AU     2.1446 eV  578.14 nm  f=0.0193  <S**2>=0.000
     119 -> 122       -0.30317
     120 -> 121        0.63737

 Excited State   5:      Triplet-AG     2.1643 eV  572.87 nm  f=0.0000  <S**2>=2.000
     119 -> 121       -0.49386
     120 -> 122        0.50375
```

[…]

S7.2. TD-B3LYP-D3: Dexcitation energies and orbital contribution in the fluorescence spectra computed for S_1

NH2T dimers 2S

136 HOMO, 137 LUMO

```
Excited State   1:      Singlet-?Sym    0.6619 eV 1873.18 nm  f=0.0000  <S**2>=0.000
     136 -> 137        0.71252
 This state for optimization and/or second-order correction.
 Total Energy, E(TD-HF/TD-DFT) =  -1608.48763629
 Copying the excited state density for this state as the 1-particle RhoCI density.

 Excited state symmetry could not be determined.
 Excited State   2:      Singlet-?Sym    1.3645 eV  908.67 nm  f=0.0001  <S**2>=0.000
     135 -> 137        0.43240
     136 -> 138        0.55885

 Excited state symmetry could not be determined.
 Excited State   3:      Singlet-?Sym    1.6177 eV  766.43 nm  f=0.0490  <S**2>=0.000
     135 -> 137        0.55989
     136 -> 138       -0.43462
```

```
 Excited state symmetry could not be determined.
 Excited State   4:      Singlet-?Sym    2.1773 eV  569.44 nm  f=0.0016  <S**2>=0.000
     135 -> 138        0.69035
     136 -> 139       -0.13164

 Excited state symmetry could not be determined.
 Excited State   5:      Singlet-?Sym    2.3467 eV  528.33 nm  f=0.0000  <S**2>=0.000
     135 -> 138        0.13232
     136 -> 139        0.69161
```

[…]

NH2T dimers 2C

136 HOMO, 137 LUMO

```
Excited State   1:      Singlet-?Sym    0.4121 eV 3008.32 nm  f=0.0000  <S**2>=0.000
     136 -> 137        0.70744
 This state for optimization and/or second-order correction.
 Total Energy, E(TD-HF/TD-DFT) =  -1608.49192033
 Copying the excited state density for this state as the 1-particle RhoCI density.

 Excited state symmetry could not be determined.
 Excited State   2:      Singlet-?Sym    1.3865 eV  894.22 nm  f=0.0150  <S**2>=0.000
     135 -> 137       -0.11290
     136 -> 138        0.70085

 Excited state symmetry could not be determined.
 Excited State   3:      Singlet-?Sym    1.8754 eV  661.09 nm  f=0.0419  <S**2>=0.000
     135 -> 137        0.69462
     136 -> 138        0.11518

 Excited state symmetry could not be determined.
 Excited State   4:      Singlet-?Sym    2.0320 eV  610.16 nm  f=0.0007  <S**2>=0.000
     136 -> 139        0.70062

 Excited state symmetry could not be determined.
 Excited State   5:      Singlet-?Sym    2.1134 eV  586.66 nm  f=0.0049  <S**2>=0.000
     136 -> 140        0.70478
```

[…]

Tetracene dimer 2S

120 HOMO, 121 LUMO

```
Excited State   1:      Singlet-?Sym    1.1247 eV 1102.34 nm  f=0.0000  <S**2>=0.000
     120 -> 121        0.71007
 This state for optimization and/or second-order correction.
 Total Energy, E(TD-HF/TD-DFT) =  -1386.93175989
 Copying the excited state density for this state as the 1-particle RhoCI density.

 Excited state symmetry could not be determined.
 Excited State   2:      Singlet-?Sym    1.9141 eV  647.75 nm  f=0.0025  <S**2>=0.000
     119 -> 121       -0.60416
```

```
    120 -> 122       -0.36695

 Excited state symmetry could not be determined.
 Excited State   3:      Singlet-?Sym    2.2932 eV  540.67 nm  f=0.0549  <S**2>=0.000
    119 -> 121       -0.36794
    120 -> 122        0.60398

 Excited state symmetry could not be determined.
 Excited State   4:      Singlet-?Sym    2.6409 eV  469.48 nm  f=0.0003  <S**2>=0.000
    118 -> 121        0.70364

 Excited state symmetry could not be determined.
 Excited State   5:      Singlet-?Sym    2.6701 eV  464.34 nm  f=0.0000  <S**2>=0.000
    117 -> 121       -0.61873
    120 -> 124       -0.33404
```

[…]

Tetracene dimer 2C

120 HOMO, 121 LUMO

```
Excited State   1:      Singlet-?Sym    1.2799 eV  968.69 nm  f=0.0000  <S**2>=0.000
    120 -> 121       -0.70852
 This state for optimization and/or second-order correction.
 Total Energy, E(TD-HF/TD-DFT) =  -1386.92759222
 Copying the excited state density for this state as the 1-particle RhoCI density.

 Excited state symmetry could not be determined.
 Excited State   2:      Singlet-?Sym    1.8674 eV  663.93 nm  f=0.0077  <S**2>=0.000
    119 -> 121        0.63908
    120 -> 122        0.30047

 Excited state symmetry could not be determined.
 Excited State   3:      Singlet-?Sym    2.2346 eV  554.85 nm  f=0.0441  <S**2>=0.000
    119 -> 121       -0.30110
    120 -> 122        0.63755

 Excited state symmetry could not be determined.
 Excited State   4:      Singlet-?Sym    2.7506 eV  450.76 nm  f=0.0000  <S**2>=0.000
    119 -> 122       -0.69736

 Excited state symmetry could not be determined.
 Excited State   5:      Singlet-?Sym    2.7811 eV  445.81 nm  f=0.0000  <S**2>=0.000
    118 -> 121       -0.62644
    120 -> 123        0.30263
```

[…]

S8. Supplementary energetic data for all the systems

In the following tables, the computed electronic, enthalpy and free Gibbs energy are reported for all the systems.

Table S6. Electronic energy, electronic energy including the zero-point energy (ZPE), enthalpy, and Gibbs free energy for all the monomers, as obtained at the (TD-)B3LYP-D3/def2-TZVP level. These energies are reported as absolute values (E, $E+ZPE$, H^0, G^0, in hartree), ZPE, H^0 and G^0 have been calculated at 1 atm and 25 °C.

	E	*E+ZPE*	H^0	G^0
**S_0**				
**T**	-693.48198726	-693.24184100	-693.22875000	-693.28016200
**NH2T**	-804.24381854	-803.96980300	-803.95363500	-804.01142000
**NMe2T**	-961.52678812	-961.14253300	-961.12025600	-961.19266000
**NPh2OMeT**	-2187.17245311	-2186.44736800	-2186.40222700	-2186.52963100
**NO2T**	-1102.65136527	-1102.40657800	-1102.38777900	-1102.45387000
**DAT**	-725.56518787	-725.34867800	-725.33591800	-725.38678100
**TrAT1**	-741.60465216	-741.39999800	-741.38738300	-741.43804300
**TrAT2**	-741.60231036	-741.39758500	-741.38494400	-741.43565400
**S_1**				
**T**	-693.39917808	-693.16118200	-693.14779100	-693.19977100
**NH2T**	-804.18559934	-803.91340500	-803.89712500	-803.95504300
**NMe2T**	-961.45704229	-961.07374900	-961.05168000	-961.12230000
**NPh2OMeT**	-2187.11332652	-2186.39036500	-2186.34502400	-2186.47334000
**NO2T**	-1102.58122420	-1102.33738900	-1102.31888900	-1102.38350400
**DAT**	-725.48620237	-725.27180000	-725.25871400	-725.31024400
**TrAT1**	-741.52789999	-741.32512700	-741.31217200	-741.36357900
**TrAT2**	-741.52584321	-741.32294100	-741.31000100	-741.36134000
**T_1 (NO TD)**				
**T**	-693.43765731	-693.20033100	-693.18698000	-693.23991600
**NH2T**	-804.21384989	-803.94152300	-803.92536500	-803.98410000
**NMe2T**	-961.48789182	-961.10529100	-961.08300900	-961.15572400

**NPh2OMeT**	-2187.13498689	-2186.41202400	-2186.36676200	-2186.49520800
**NO2T**	-1102.61096060	-1102.36818200	-1102.34941500	-1102.41595900
**DAT**	-725.52072341	-725.30694500	-725.29393400	-725.34567000
**TrAT1**	-741.56014792	-741.35806200	-741.34519100	-741.39740600
**TrAT2**	-741.55928838	-741.35708000	-741.34420700	-741.39641700

Table S7. Electronic energy, electronic energy including the zero-point energy (ZPE), enthalpy, and Gibbs free energy for all the dimers, as obtained at the (TD-)B3LYP-D3/def2-TZVP level. These energies are reported as absolute values (E, $E+ZPE$, H^0, G^0, in hartree), ZPE, along with the BSSE. H^0 and G^0 have been calculated at 1 atm and 25 °C. These energies are reported as absolute values (E, H^0, G^0, in hartree). H^0 and G^0 have been calculated at 1 atm and 25 °C. For T_{1-A}, because of the difficulties in defining a not-integer spin state for each monomer, the BSSE values obtained for T_{1-B} have been used to determine the energetic values reported in Table 3.

	E	$E+ZPE$	H^0	G^0	BSSE
**S_0**					
**Tetracene-2S**	-1386.992269	-1386.510377	-1386.483419	-1386.565606	0.001434693
**Tetracene-2C**	-1386.990288	-1386.508495	-1386.48147	-1386.563915	0.001305462
**NH2T-2S**	-1608.526402	-1607.975995	-1607.94324	-1608.036219	0.002658112
**NH2T-2C**	-1608.524177	-1607.973714	-1607.940993	-1608.034461	0.002592117
**S_1**					
**Tetracene-2S**	-1386.93175989	-1386.45159100	-1386.42466500	-1386.50545700	0.001461377
**Tetracene-2C**	-1386.92759222	-1386.44753300	-1386.42052000	-1386.50239400	0.001425399
**NH2T-2S**	-1608.48763629	-1607.93884400	-1607.90649400	-1607.99772300	0.00300387
**NH2T-2C**	-1608.49192033	-1607.94141400	-1607.90954900	-1608.00043300	0.002894169
**T_{1-A}**					
**Tetracene-2S**	-1386.93490394	-1386.45471000	-1386.42763900	-1386.51055600	–
**Tetracene-2C**	-1386.93490393	-1386.45471100	-1386.42763900	-1386.51055600	–
**NH2T-2S**	-1608.48688977	-1607.93704300	-1607.90457800	-1607.99727000	–
**NH2T-2C**	-1608.48741758	-1607.93699000	-1607.90475600	-1607.99709800	–
**T_{1-B}**					
**Tetracene-2S**	-1386.94556072	-1386.46682800	-1386.43937000	-1386.52688400	–
**Tetracene-2C**	-1386.94685499	-1386.46777500	-1386.44047700	-1386.52490700	–

**NH2T-2S**	-1608.49449190	-1607.94601700	-1607.91308200	-1608.00881900	–
**NH2T-2C**	-1608.49432796	-1607.94543400	-1607.91278000	-1608.00685000	–
**Q_1**					
**Tetracene-2S**	-1386.90121064	-1386.42508800	-1386.39739900	-1386.48372400	0.00125717
**Tetracene-2C**	-1386.90143163	-1386.42526600	-1386.39769000	-1386.48289600	0.00137104
**NH2T-2S**	-1608.46245779	-1607.91581800	-1607.88280300	-1607.97916400	0.00261009
**NH2T-2C**	-1608.46039811	-1607.91389800	-1607.88091600	-1607.97666600	0.00260696

S9. Coordinates of all the optimized structures

Cartesian coordinates of all the structures discussed in the article are reported in the xyz format in the Supporting Information. They are also available at the Zenodo repository with DOI: 10.5281/zenodo.17574963.

References

1. Yu, D.; Stuyver, T.; Rong, C.; Alonso, M.; Lu, T.; De Proft, F.; Geerlings, P.; Liu, S., Global and local aromaticity of acenes from the information-theoretic approach in density functional reactivity theory. *Phys. Chem. Chem. Phys.* **2019,** *21* (33), 18195-18210.
2. Patra, S. G.; Mondal, H.; Bhattacharjya, M. J.; Chetia, N.; Chattaraj, P. K., On the aromaticity of substituted benzene. *Theor. Chem. Acc.* **2023,** *142* (10), 91.
